# How the Fly Balances Its Ability to Combat Different Pathogens

**DOI:** 10.1371/journal.ppat.1002970

**Published:** 2012-12-13

**Authors:** Moria C. Chambers, Karla L. Lightfield, David S. Schneider

**Affiliations:** Department of Microbiology and Immunology, Stanford University, Stanford, California, United States of America; University of Minnesota, United States of America

## Abstract

Health is a multidimensional landscape. If we just consider the host, there are many outputs that interest us: evolutionary fitness determining parameters like fecundity, survival and pathogen clearance as well as medically important health parameters like sleep, energy stores and appetite. Hosts use a variety of effector pathways to fight infections and these effectors are brought to bear differentially. Each pathogen causes a different disease as they have distinct virulence factors and niches; they each warp the health landscape in unique ways. Therefore, mutations affecting immunity can have complex phenotypes and distinct effects on each pathogen. Here we describe how two components of the fly's immune response, melanization and phagocytosis, contribute to the health landscape generated by the transcription factor ets21c (CG2914) and its putative effector, the signaling molecule wntD (CG8458). To probe the landscape, we infect with two pathogens: *Listeria monocytogenes*, which primarily lives intracellularly, and *Streptococcus pneumoniae*, which is an extracellular pathogen. Using the diversity of phenotypes generated by these mutants, we propose that survival during a *L. monocytogenes* infection is mediated by a combination of two host mechanisms: phagocytic activity and melanization; while survival during a *S. pneumoniae* infection is determined by phagocytic activity. In addition, increased phagocytic activity is beneficial during *S. pneumoniae* infection but detrimental during *L. monocytogenes* infection, demonstrating an inherent trade-off in the immune response.

## Author Summary

The importance of individual immune responses is incredibly infection dependent, and this paper harnesses the variability in two mutant lines to explain the relative importance of two aspects of fly immunity: melanization and phagocytosis. Increased phagocytic activity is beneficial during *S. pneumoniae* infection due to increased clearance of the extracellular microbe and detrimental during *L. monocytogenes* infection as it increases the intracellular niche for *L. monocytogenes*. Outcomes during *L. monocytogenes* infection are also dependent on melanization capability, which impacts the ability to control extracellular bacteria.

## Introduction

Infected fruit flies get sick in ways that human patients would recognize; bacterial infections in *Drosophila* induce changes in feeding, metabolism and circadian rhythm, and conversely changes in these pathways influence susceptibility to infection [1]–[3]. Many responses affect survival during infection, but this work remains splintered as the field primarily focuses on individual mechanisms in isolation, offering glimpses of the whole picture. Here we use mutations in two genes, ets21c and wntD, to examine their effect on immune responses and survival during infections with two bacteria, *Listeria monocytogenes* and *Streptococcus pneumoniae*. Both genes affect multiple arms of the immune system and we wanted to understand how immunity offers protection against pathogens with different lifestyles in *Drosophila*. We chose these two microbes because they produced dissimilar phenotypes in previous *Drosophila* immunity assays [4]. Together these mutants and microbes demonstrate how there can be no perfect immune response, as there are responses that are beneficial during one infection and actively detrimental during another.

The *Drosophila* immune response can be divided into categories based on the speed at which they act following pathogenic challenge. The fast-acting immune responses, which respond within seconds to minutes, are phagocytosis and melanization [5]. Hemocytes are phagocytic cells in the fly and they are concentrated in adherent groups on the dorsal side of the abdomen and the anterior abdominal segment of the heart in adult flies. Inhibition of phagocytosis increases susceptibility to a number of bacteria [6]–[9]. Insects produce melanin from tyrosine using the enzyme phenoloxidase, which is activated by an immune triggered proteolytic cascade. This process is hypothesized to produce reactive oxygen species, which can harm the host in addition to harming the pathogen, and to physically encapsulate the invaders [10], [11]. In *Drosophila*, some bacterial pathogens (*L. monocytogenes*, *Salmonella typhimurium*, and *Staphylococcus aureus*) induce visible melanization, and flies defective in the melanization activation pathway are less resistant to these infections [4]. Though these relatively quick responses presumably remain active through the whole infection there is at least one response that takes several hours to reach full force. This slow response is the induction of anti-microbial peptides which peak in transcript expression six to 24 hours post infection [12]. We do not know when actual antimicrobial activity peaks as this is seldom assayed directly, but presumably this takes even longer than the increase of transcripts.

While it may be simplest to examine the effect of immune components individually, in order to effectively control immunity clinically we need a better understanding of the full immune network; each response doesn't exist in a vacuum. Knowing which immune responses strongly associate with a positive outcome for a given pathogen and which physiological systems are impacted by infection will allow doctors to more effectively treat disease. Patients normally do not have a single pathway or gene responsible for their entire pathology, and we need to develop the tools to deal with these levels of complexity.

To probe changes in the immune response, we turned to two pathogens that previously exhibited opposing phenotypes: *Listeria monocytogenes* and *Streptococcus pneumoniae*. When injected into the hemocoel, *L. monocytogenes* causes lethal infections in *Drosophila melanogaster* at doses as low as ten bacteria, and death from infection occurs on the order of one week. *L. monocytogenes* lives both intracellular and extracellular in the fly and causes robust disseminated melanization [4], [13]–[15]. *S. pneumoniae* can also cause lethal infections; however, there are sub lethal doses, which prime the fly to become resistant upon subsequent challenges [16]. *S. pneumoniae* infection kills flies rapidly, within two to four days, and flies surviving past four days have likely cleared the pathogen. *S. pneumoniae* is an extracellular pathogen and bead inhibition of phagocytosis increases susceptibility to infection [6]. In contrast to *L. monocytogenes*, flies deficient in melanization are more resistant to S. *pneumoniae* infection, although the mechanism is unknown [4].

Ets21c (CG2914), a putative transcription factor characterized by its DNA binding ets-domain, was previously implicated in *Drosophila* immunity. Ayres et al. found that et21c mutants died more rapidly during *L. monocytogenes* infection with similar bacterial loads compared to wild-type, but were no different from wild type flies when challenged with *S. typhimurium* or *S. aureus* [17]. Studies of immune signaling in *Drosophila* S2 cells and hemocyte cell lines used ets21c transcript as a read out of the early immune response and showed that ets21c induction depends on the imd pathway and one of its transcription factors, basket [18], [19].

WntD (CG8458) is a negative regulator of dorsal signaling in *Drosophila*, and wntD mutants are more susceptible to *L. monocytogenes* infection than wild type flies [20]. Previous work measured the signaling and transcriptional effects of wntD on antimicrobial peptides [21]; however, it remains unknown, whether wntD impacts bacterial load during *L. monocytogenes* infection and how it affects melanization and phagocytosis.

The effect of a given bacterial load has previously been used to categorize genes as either impacting tolerance or resistance [2], [4], [17]. Resistance genes and mechanisms directly impact how well the bacteria grow or are killed, while tolerance genes and mechanisms affect the host's ability to deal with the effect of infection (e.g. energy strain, accumulated damage). While both of these mechanisms are functionally distinct, they way they impact bacterial load cannot be as easily separated and there is a full spectrum of phenotypes possible, from genes that do not impact bacterial load at all to genes that increase bacterial load by hundred-fold in just a day. Determining where in this spectrum our mutants fall helps inform the possible responsible mechanisms.

In this paper, we show that ets21c and wntD mutants are both more susceptible to *L. monocytogenes* and more resistant to *S. pneumoniae*, but differ in their ability to control *L. monocytogenes* bacterial loads. At the levels of specific immune responses, these mutants share an increase in phagocytic activity and a shift in anti-microbial peptide induction, but differ in their melanization capabilities. By examining these differences, we establish the relative contributions of the immune pathways to these outcomes - survival during *L. monocytogenes* infection depends on multiple factors: melanization and phagocytic ability while phagocytic ability alone predicts survival to *S. pneumoniae* infection.

## Results

### Ets21c mutants do not induce wntD during *L. monocytogenes* infection

**Figure 1:**
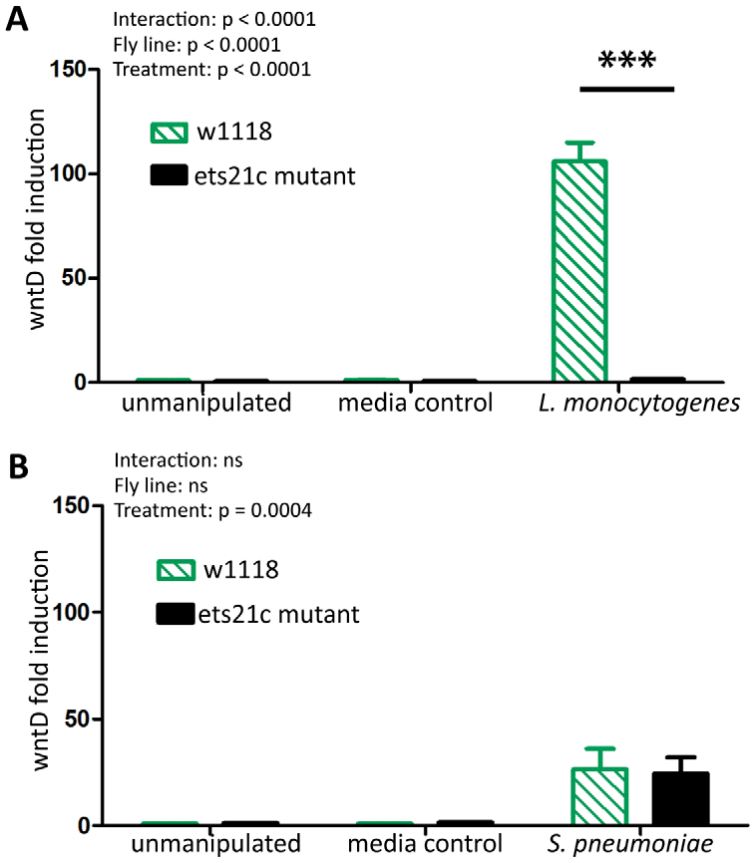
Ets21c mutants do no induce wntD during infection. WntD expression in ets21c mutants six hours after both *L. monocytogenes* (A) and *S. pneumoniae* (B) infection as assayed by qRT-PCR. The significant sources of variation were assessed by two-way ANOVA and differences in wntD expression between fly lines during each treatment were assessed by the Bonferroni post-test after ANOVA and significantly different values denoted by asterisk (*** p<0.001).

Ets21c is a putative transcription factor therefore we assessed the impact of an ets21c mutation on the transcriptome by performing a microarray analysis on infected flies. Complete microarray data is available in the online supplemental materials (Dataset S1). A familiar gene emerged in our list of infection induced genes in the parental line: wntD. WntD is a negative regulator of the dorsal pathway and wntD mutants die more quickly during *L. monocytogenes* infections [20]. Ets21c mutant flies do not upregulate wntD during *L. monocytogenes* infection and we confirmed this using real-time qRT-PCR (Figure 1A, p<0.001). *S. pneumoniae* induces expression of wntD in both the ets21c mutant and its parental line, but only 25-fold, which is lower relative to the hundred-fold induction during *L. monocytogenes* infection. The reciprocal effect of wntD mutants on ets21c expression was examined both in the microarray published by Gordon et al. and by qRT-PCR, but the levels of ets21c were so low in total fly RNA preparations that results were highly variable and therefore not significant (data not shown). WntD is a good candidate effector for ets21c's immune phenotypes, due to wntD's ability to impact survival to *L. monocytogenes*.

### Ets21c and wntD impact susceptibility to *Listeria monocytogenes* differently

**Figure 2:**
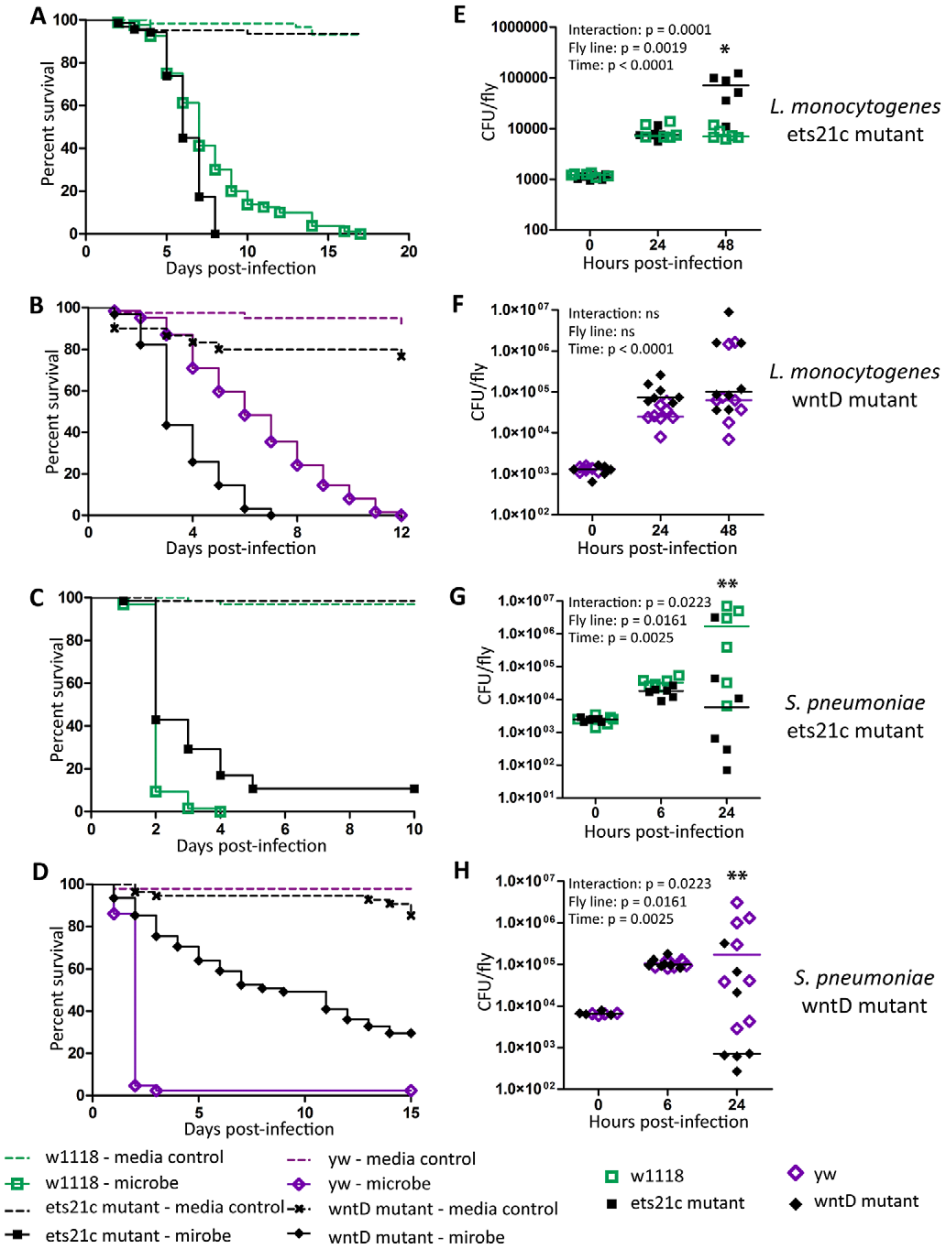
Ets21c and wntD similarly affect survival to *L. monocytogenes* and *S. pneumoniae.* (A–D) Bacteria were injected into flies and the flies were monitored for survival (A),(B) *L. monocytogenes* OD_600_ = 0.01 (C) *S. pnuemoniae* OD_600_ = 0.05 (D) *S. pneumoniae* OD_600_ = 0.2. Log-rank analysis of the survival curves gives p<0.0001 (w/o media controls in analysis). (E–H) Bacteria was injected into flies and the flies and CFUs monitored at various time points post injection (E),(F) *L. monocytogenes* OD_600_ = 0.01 (G) *S. pnuemoniae* OD_600_ = 0.05 (H) *S. pneumoniae* OD_600_ = 0.2. The significant sources of variation were assessed by two-way ANOVA and differences in bacterial load between fly lines at each time point were assessed by the Bonferroni post-test after ANOVA and significantly different values denoted by asterisk (* p<0.05, ** p<0.01).

**Figure 3:**
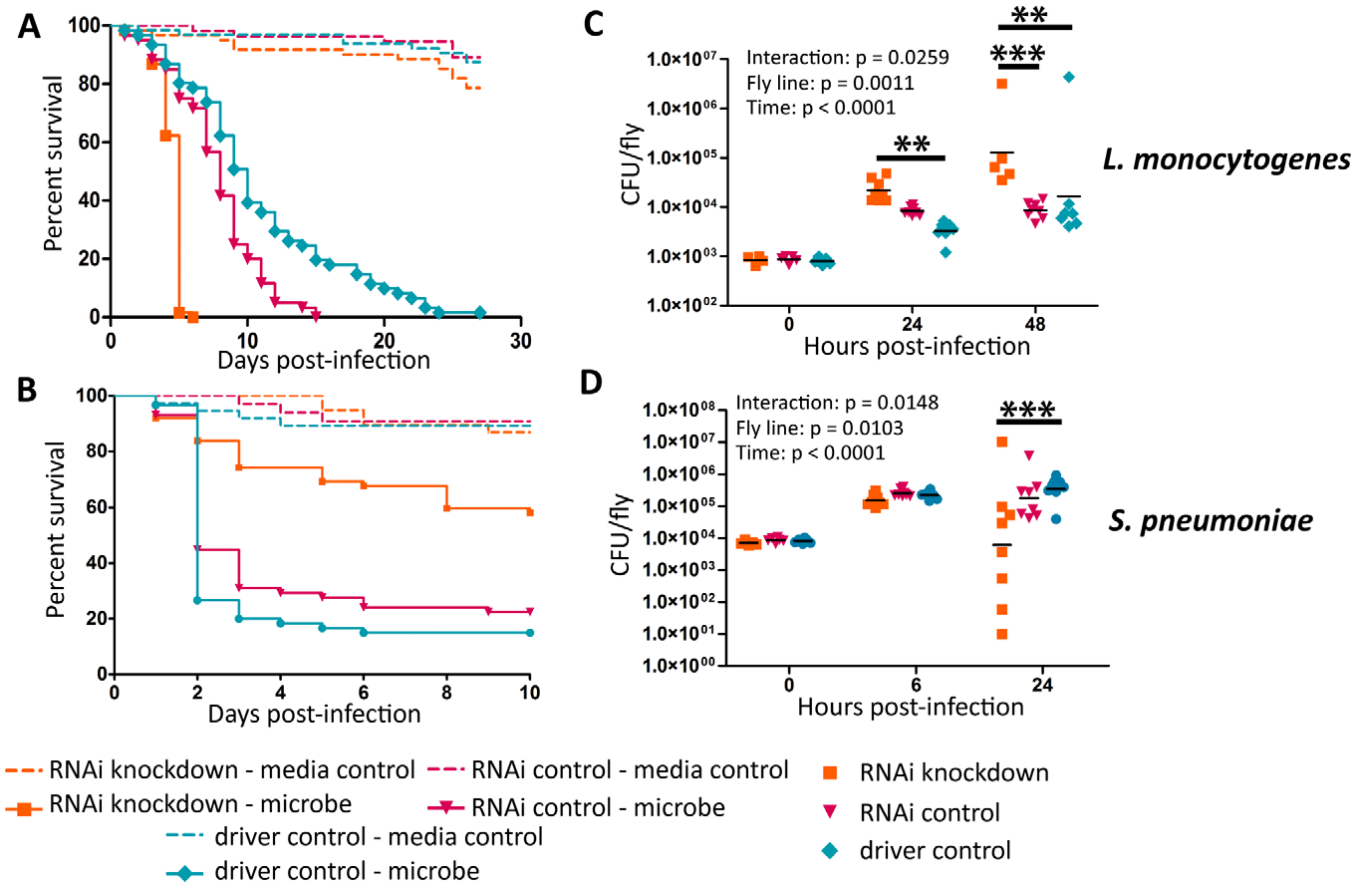
Ets21c phenotypes confirmed by RNAi knockdown. *L. monocytogenes* or *S. pneumoniae* were injected into RNAi crosses and control flies. Survival and growth of the bacteria was monitored over the course of infection. (A),(C) L. monocytogenes; (B),(D) S. pneumoniae. Log-rank analysis of the survival curves gives p<0.0001 for all curves (w/o media controls in analysis). The significant sources of variation were assessed by two-way ANOVA and differences in bacterial load between the driven RNAi and the controls at each time point were assessed by the Bonferroni post-test after ANOVA and significantly different values denoted by asterisk (** p<0.01, *** p<0.001).

Ayres and colleagues published that ets21c mutants have increased susceptibility to *L. monocytogenes* with no significant increase in bacterial load causing them to conclude that the gene affected tolerance [17]. This mutant did, however, show an insignificant increase in *L. monocytogenes* bacterial load two days post-infection. Upon retesting, we found that these mutants exhibit a small but significant increase in bacterial load at 48 hours post infection (Figure 2A,E). We call this a small effect since there is no change at 24 hours and a nine fold increase in bacterial load at 48 hours whereas a mutation in another gene, gr28b, increases bacterial growth 100 fold at both 24 and 48 hours. This relatively small increase in bacterial growth rates in ets21c mutants was confirmed with flies that had ets21c expression knocked down by RNAi in the fatbody (Figure 3 A,B). Gordon and colleagues showed that wntD mutants were more susceptible to *L. monocytogenes*, but did not report bacterial loads [20]. We confirmed that wntD mutants die faster than parental flies (Figure 2B), and found that wntD mutants remain able to control bacterial growth to at least 48 hours post-infection (Figure 2F). We stopped measuring CFU at this point as over half the mutant flies die the next day and we worried that we would skew results if we were looking at survivors that may be more resistant or potentially received a smaller infectious dose. Knockdown of wntD in the fatbody confirmed wntD's effect on tolerance to *L. monocytogenes* (Figure S1). Mutants in ets21c and wntD are both more susceptible to *L. monocytogenes* infection, but fall on different parts of the tolerance-resistance continuum indicating that there may be mechanistic difference at play in these lines.

### Sensitivity to *Listeria monocytogenes* correlates with RNAi driver strength in the fatbody

*Drosophila* has an excellent tool for knocking down gene expression in a tissue specific manner; tissue specific expression of the transcription factor GAL4 can be used to drive gene specific RNAi constructs. A large number of these tissue specific GAL4 lines and RNAi lines are publically available and one simply has to cross the driver line to the RNAi line and test the appropriate offspring. However, these lines are not perfectly tissue specific and can have significant expression levels in a variety of tissues. We tested a panel of GAL4 drivers to determine where ets21c was required during infection and found it difficult to interpret the data because all drivers tested produced a similar phenotype (Figure S2).

**Figure 4:**
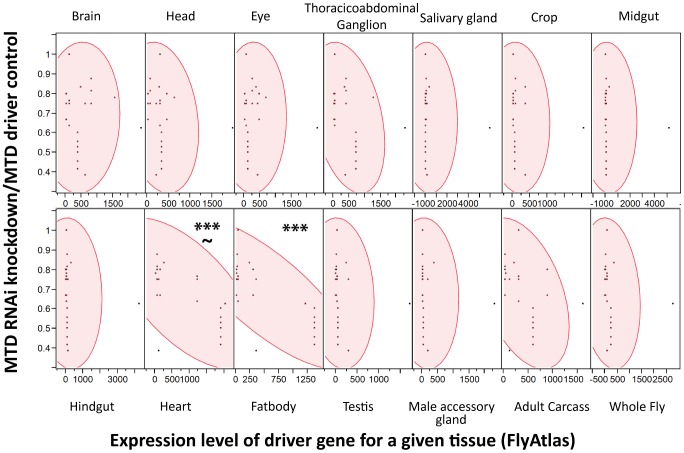
Strength of RNAi induced *L. monocytogenes* phenotype correlates with driver expression in the fatbody. Scatterplot matrices were generated with JMP software for each tissue using expression levels for each driver gene within the tissue from FlyAtlas and a measure of the immune phenotype for that driver(median time to death RNAi×Driver/MTD w1118×Driver). Significance was determined by ANOVA (*** p<0.0001), and the test Lack of Fit used to assess the likely hood that the linear model was poor (∼p<0.01).

We reasoned that the problem was that driver localizations are primarily determined by ability to drive GFP expression in tissues; however, we worried that low expression levels of an RNAi construct might be sufficient to produce a phenotype while registering as background in a fluorescent microscopy assay that measured the induction of GFP. Instead of using the published localization for each driver, we assumed that the driver strength matched the expression data for each driver gene as reported by FlyAtlas, a database of tissue specific gene expression results from both larvae and adults. For each tissue, we used JMP Software (http://www.jmp.com) to determine whether there was a significant correlation between the expression level of the driver gene with the strength of immune phenotype as measured by median time to death (MTD) of RNAi×Driver/MTD of the driver control. As shown in Figure 4, higher expression of the driver genes in both the heart and fatbody correlated with increased sensitivity to *L. monocytogenes* infection. However, correlation of sensitivity to infection and driver strength in the heart was not as strong and also had a significant p-value for the lack of fit test indicating that the linear model may not be appropriate for this tissue. Driver expression values in the heart correlate with those in the fatbody (data not shown), so it is unsurprising that they would both exhibit a similar relationship with the sensitivity to *L. monocytogenes* infection. This data does not rule out a role for hemocytes as they are not reported in FlyAtlas and could potentially have adhered to other tissues, particularly the heart, during the isolation used for the FlyAtlas data. The closest available approximation for adult hemocytes was the expression data available for growing S2 cells in culture, which are known to have phagocytic properties, and microarray data published on larval hemocytes. Both of these cultured cells revealed a significant correlation between strength of driver expression and sensitivity to *L. monocytogenes* infection by ANOVA, but also had significant p-values for the lack of fit test indicating that the model may be inappropriate (data not shown). While it is unsurprising that the fatbody and/or hemocytes are important for our immune phenotype, this technique can be broadly applied to any phenotype that can be quantified and will allow quantitative and methodical use of drivers.

### Ets21c and wntD both impact resistance to *Streptococcus pneumoniae*

Ets21c mutants had been previously shown to have no impact during *S. typhimurium* or *S. aureus* infection, while published studies on wntD mutants only examined the impact during *L. monocytogenes* infection [17], [20], [21]. We chose to further test the specificity of ets21c with *Streptococcus pneumoniae* because it behaves uniquely in some other *Drosophila* immunity mutants [4], [22]. Mutants in both ets21c and wntD survive *S. pneumoniae* challenge better (Figure 2C–D) and with decreased bacterial loads compared to parental strains (Figure 2G–H). This phenotype was also confirmed using RNAi knockdown in the fatbody as described above (Figure 3B,D and Figure S1B,D). These results indicate that mutants in ets21c and wntD both have an increased resistance to *S. pneumoniae* infection, whereas they exhibited differences in their type of susceptibility to *L. monocytogenes*.

### WntD mutants have more powerful fast-acting immune responses

**Figure 5:**
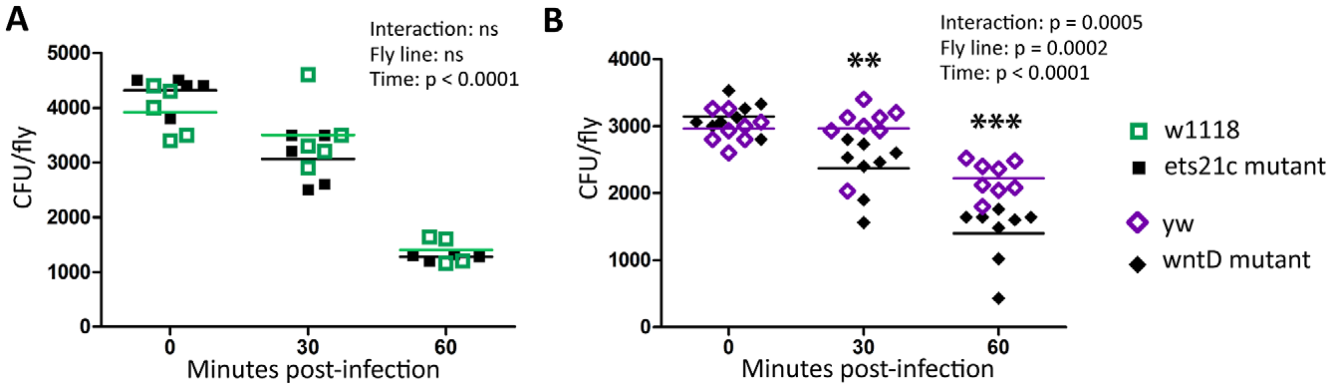
WntD mutants have increased ability to clear *E. coli.* Ets21c mutant (A) and wntD mutant (B) flies were injected with *E. coli* and CFUs determined on a quick time-course to assess differences in fast acting immune responses. The significant sources of variation were assessed by two-way ANOVA and differences in bacterial load between fly lines at each time point were assessed by the Bonferroni post-test after ANOVA and significantly different values denoted by asterisk (** p<0.01, *** p<0.001).

To examine which immune responses are responsible for the differences between ets21c and wntD mutants, we performed assays of each immune pathway to assess the strength of both fast acting and slow acting immune responses. Melanization and phagocytosis begin to act within seconds to minutes of infection, while anti-microbial peptide induction takes hours. It can be difficult to measure the strength of immune responses against pathogens; pathogens often evolve mechanisms to beat the immune response. To assess the strength of the fast acting responses we injected flies with *Escherichia coli*, which is non-pathogenic to *D. melanogaster* at this dose and is rapidly cleared from circulation. By focusing on the first hour post-injection, we assayed the fast acting responses of phagocytosis and melanization. While this is a very different microbe from both *L. monocytogenes* and *S. pneumoniae*, basic immune responses are likely conserved between infections and this is a first approximation of fast-acting responses. Ets21c mutants do not clear *E. coli* more quickly (Figure 5A). WntD mutants, however, have significantly increased *E. coli* clearance (Figure 5B). This result does not distinguish whether shifts in melanization or phagocytosis are occurring, and does not rule out the possibility that ets21c mutants have shifts in both melanization and phagocytosis for an overall neutral effect on *E. coli* clearance.

### Ets21c and wntD mutants have increased phagocytic activity

**Figure 6:**
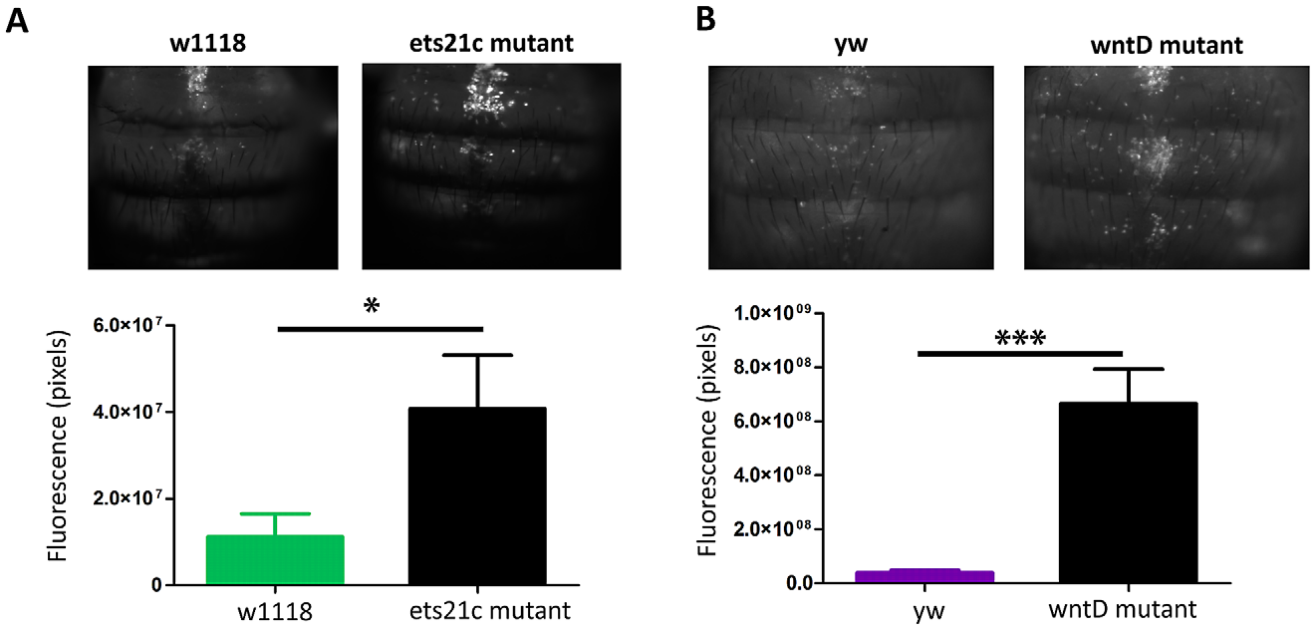
Ets21c and wntD mutants both have increased phagocytic activity relative to their parental line. pHrodo labeled *E. coli* were injected into flies and flies were imaged after 30 minutes. Ets21c mutant and w1118 flies imaged for 1600 ms (A); WntD mutant and yw flies imaged for 800 ms(B). Significance was determined by a two tailed t-test (* p<0.05; ***p<0.001).

To determine the potential contribution of each of the fast acting immune responses to the observed phenotypes, we wanted to test both phagocytic ability and melanization capabilities separately. We determined the strength of phagocytic ability by assaying the ability of our mutants to phagocytose dead bacteria. We imaged flies injected with a pHrodo labeled *E. coli*, which only fluoresces upon encountering a low pH like that found in phagosomes; while pHrodo labeled *L. monocytogenes* would be the more perfect tool it isn't commercially available and the labeled *E. coli* provides an initial approximation for phagocytic ability. Quantifying the amount of fluorescence revealed that each mutant phagocytosed more bacteria than their parental line (Figure 6). There was also a dramatic difference in the phagocytic activity of the two parental lines, as evidenced by the fact that we had to use twice as long an exposure to take images of w1118 and ets21c mutants as compared with yw and wntD mutants. No direct comparison between all four lines was done because exposures which can capture w1118 and ets21c mutant differences completely over-exposes the other lines. These four lines offer a spectrum of phagocytic abilities giving us a broad dynamic range to assess the importance of phagocytosis during infection.

### Increases in phagocytic ability are associated with an increase of intracellular *L. monocytogenes*

**Figure 7:**
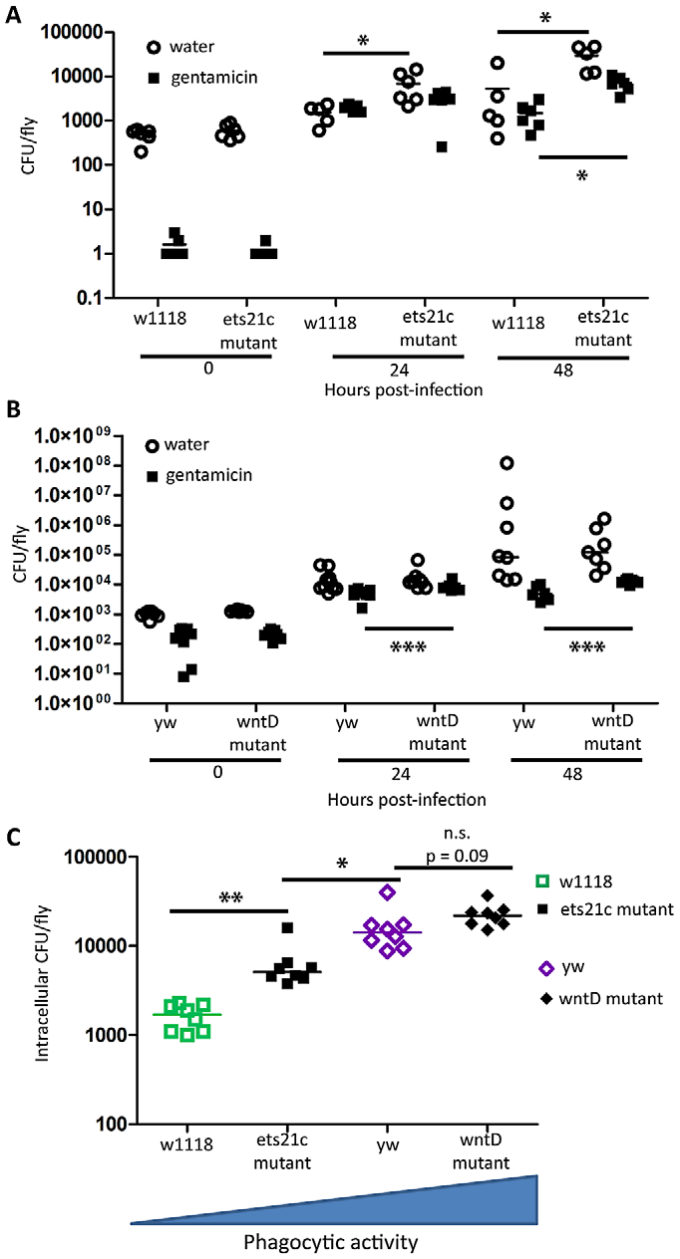
Increased phagocytic activity associated with increased intracellular *L. monocytogenes.* (A),(B) Flies were injected *L. monocytogenes* and at various time-points after infection intracellular and total populations were assessed by gentamicin chase. Significance was determined by a two tailed t-test (* p<0.05; ** p<0.01,***p<0.001). (C) Intracellular population as determined by gentamicin condition, 48 hours post-injection. Significance was determined by a one-tailed t-test (*p<0.05, ** p<0.01).

*L. monocytogenes* is a facultative intracellular bacterium that is capable of escaping the phagosome and living within the cytosol of phagocytic cells [15], [23], [24]. While an increase in phagocytosis may help clear extracellular pathogens such as *S. pneumoniae*, this increase may give additional access to a niche for *L. monocytogenes*. We tested this by determining how many intracellular *L. monocytogenes* were found in infected flies. Gentamicin is an antibiotic that is unable to cross cell membranes and injecting this antibiotic into the fly's circulation allows us to measure intracellular and extracellular populations. Figure 7 shows that both wntD and ets21c mutants have increased intracellular bacterial loads compared with their parental line. The water injected control, which reports total bacteria, further confirms that ets21c mutants also have an overall increase in bacterial load at 48 hours post-infection while wntD mutants have no significant change. When comparing the intracellular populations for all four lines at once, we noticed that the intracellular population was positively associated with phagocytic ability (Figure 7C).

### Ets21c and wntD have opposite effects on melanization

**Figure 8:**
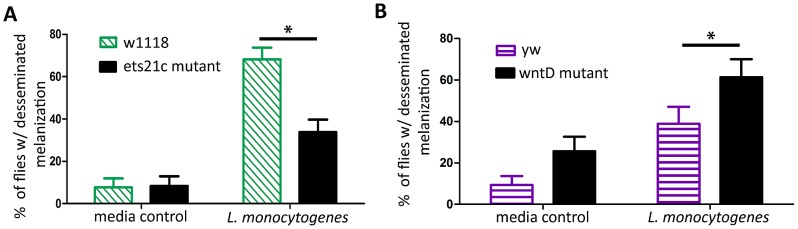
Melanization is reduced in ets21c mutants and increased in wntD mutants. Flies were tested for their ability to melanize during infection by looking for disseminated melanization 4 days post-injection (A) or 3 days post-injection (B) Significance was determined by Fischer's Exact Test (* p<0.05).

We assayed the second fast immune response, melanization, by looking at the capability of the flies to show disseminated melanization after infection. *L. monocytogenes* causes visible melanotic spots within 3–4 days after infection, and flies defective in melanization are more susceptible to infections that cause this disseminated melanization [4]. When flies were scored as positive or negative for melanization, a significantly lower proportion of ets21c mutants exhibited visible disseminated melanization (Figure 8A), while a higher proportion of wntD mutants exhibited disseminated melanization (Figure 8B).

### Ets21c affects anti-microbial peptide transcription similarly to wntD

**Figure 9:**
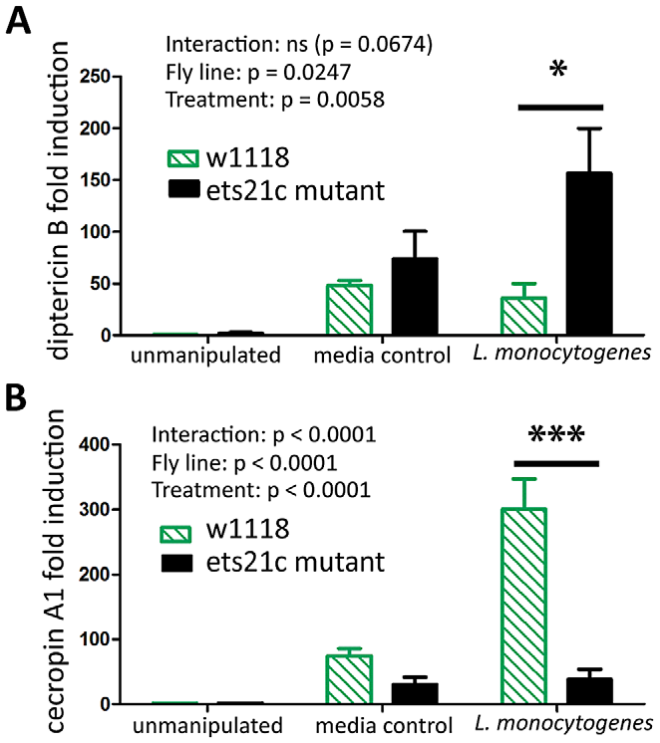
Ets21c has a minor affect on anti-microbial peptide expression similar to that of wntD. Antimicrobial peptide expression in ets21c mutant six hours after *L. monocytogenes* infection. (A) diptericin B (B) cecropin A. The significant sources of variation were assessed by two-way ANOVA and differences in anti-microbial peptide expression between fly lines during each treatment were assessed by the Bonferroni post-test after ANOVA and significantly different values denoted by asterisk (** p<0.01, *** p<0.001).

We examined anti-microbial peptide (AMP) gene induction in these two mutants. Gordon et al. reported that wntD mutants had increased induction of diptericin upon *L. monocytogenes* infection but saw no change in the induction of drosomycin [20]. We also found that ets21c mutants also have a four-fold increased induction of diptericin (p<0.05)(Figure 9A) and no change in drosomycin induction (data not shown). We also tested attacin, metchnikowin, defensin, drosocin and cecropin for changes during *L. monocytogenes* infection and found that ets21c only affected cecropin expression in that it was poorly induced during infection, about 10-fold lower than its parental line (p<0.001) (Figure 9B). In the microarray, ets21c mutants also showed up-regulation of most anti-microbial peptides, and did not show significantly different induction than the parental line (Figure S3). We suggest that this is a minor but complex impact on anti-microbial peptide expression as the majority of transcripts do not change and when they do change they can go up or down. While individual anti-microbial peptides have been shown to impact survival to infection in *Drosophila*, their effect was only visible in an otherwise immune compromised mutant with forced high expression of the anti-microbial peptide [25]. We do not understand the impact of modest changes in AMPs in a background where many AMPs are highly expressed and are unchanging.

## Discussion

**Figure 10:**
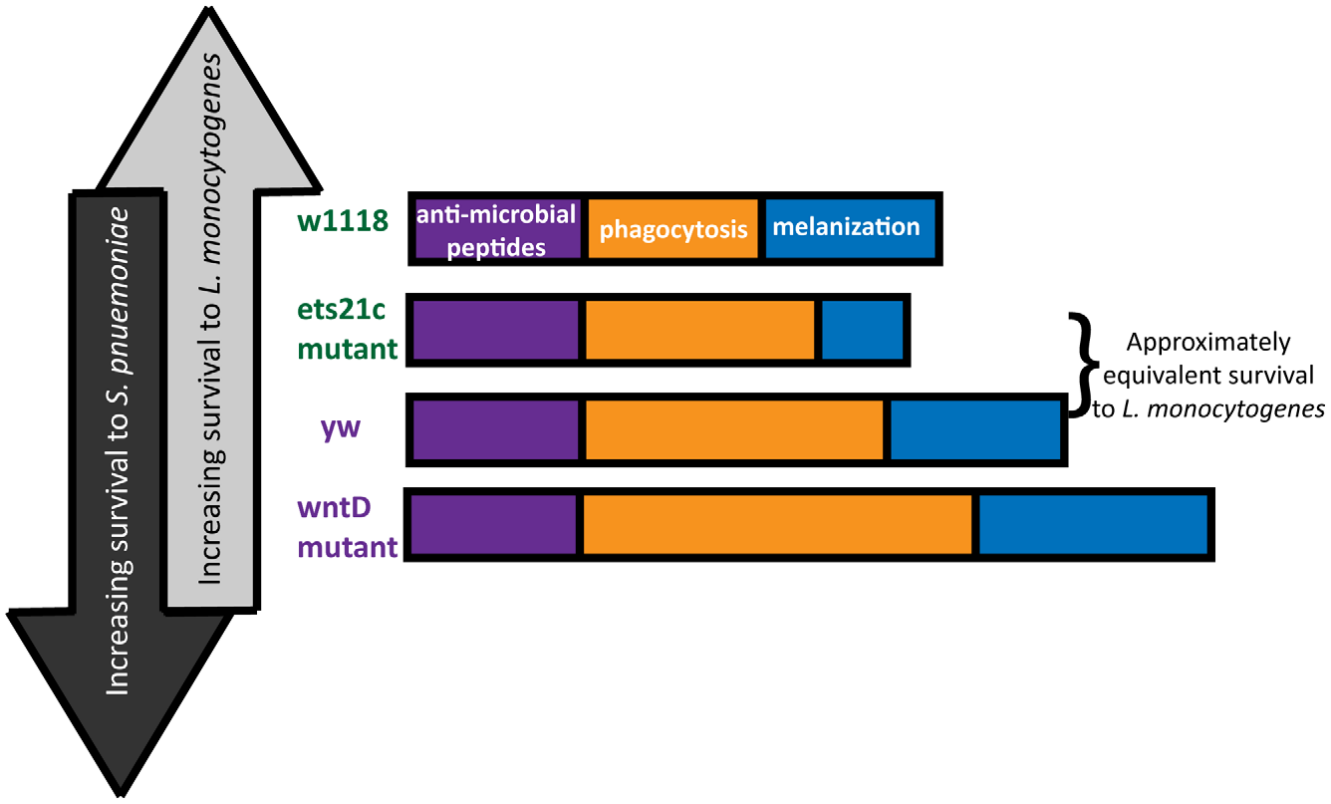
Building an immune response for antagonistic purposes. Fly lines are arranged in order of both *L. monocytogenes* and *S. pneumoniae* susceptibility. The “strength” of each resistance response is represented by the length of the bars.

The diverse and often opposing strengths and weaknesses of different pathogens leads to inherent trade-offs in immunity. In order to observe these trade-offs, one must infect with a range of pathogens and explore multiple arms of the immune response. This research used mutants in two genes to explore the contribution of two immune components: phagocytosis and melanization to the survival during infections with two bacteria: *L. monocytogenes* and *S. pneumoniae*. The line most resistant to *S. pneumoniae* dies the fastest when faced with *L. monocytogenes* and the reciprocal also holds true (Figure 10). The differences between these pathogens make them useful tools with which to probe the immune system.

This paper focuses primarily on the immune contribution of the fly's phagocytic ability. Due to differences in pathogen lifestyle, the increased phagocytosis in our mutants has unique consequences for each bacterium. *S. pneumoniae* is an extracellular bacterium, and phagocytosis is a death knell. *L. monocytogenes* can harness phagocytosis as an entry way to a protected niche. Our work shows that flies with the most phagocytosis are most susceptible to *L. monocytogenes* and have the correspondingly highest intracellular populations of *L. monocytogenes* while also being most resistant to *S. pneumoniae*. This presents a perplexing dilemma for the design of a robust immune system – what is the most advantageous amount of phagocytosis? This will depend on the frequency of pathogens encountered that will take advantage of this potential niche.

While it might be tempting to explain the range of phenotypes simply by the amount of phagocytic ability, the approximately equivalent survival of the ets21c mutant and yw parental line in spite of a difference in phagocytic ability suggests that additional factors might be at work. A second ingredient, melanization, is potentially that additional factor. The differential impact that ets21c and wntD have on melanization offers an explanation for why ets21c affects resistance while wntD affects tolerance to *L. monocytogenes*. Too little melanization is detrimental during a *L. monocytogenes* infection and causes increased extracellular bacteria [4]. Ets21c mutants decrease, but do not obliterate of the ability to melanize and have a corresponding significant but mild difference in their resistance to *L. monocytogenes*. WntD mutants, however, have an increased proportion of flies showing melanization compared to their parental line. The effects of hyper-melanization during infection are unknown, but production of melanin results in the production of reactive oxygen species (ROS) which can potentially harm the host as well as the microbe. If this increase in melanization in WntD mutants harms the host through ROS production, it could help explain the mutant's defect in tolerance to *L. monocytogenes*. The flies may become more dependent on this immune response and cause futile damage in their efforts to contain the bacteria.

Our results relegate anti-microbial peptides to a supporting role, primarily because we saw transcriptional changes in very few AMPs. Knockout of a single AMP has never been reported to produce a survival phenotype and we observed both increases and decreases of individual AMP induction. However, our data does not rule out the possibility that there is an AMP which can specifically affect either *S. pneumoniae* or *L. monocytogenes* and influence the phenotype. We believe this is unlikely because of the negative association between the two phenotypes and over-expression of a single AMP would have to be capable of producing the opposite effect on the other bacteria.

Another potential factor that this paper does not directly address is energy investment. Implicit in a stronger response is the energetic cost of mounting that response. While we do not measure this cost in our mutants, this is still a factor that could be influencing their survival outcome, especially of wntD mutants. These flies have a hyper-melanization response and increased phagocytic ability which may restrict the fly's access to a crucial amino acid – tyrosine, which is the precursor for melanin production. This could be a contributing factor to why these flies die so quickly compared to the other lines, while having the “strongest” of each of the immune responses.

A robust immune system must have an appropriate balance of immune responses to account for the diversity of pathogens it will encounter; however, even a well designed immune system will contain tradeoffs. A better appreciation of the natural and inevitable antagonism will help us gain a more in-depth appreciation for the evolutionary history behind our immune systems. Encouraging scientists to embrace pathogens which reveal distinct and even opposite phenotypes is necessary to fully explore the robustness and complexities of the immune response.

## Materials and Methods

### *D. melanogaster* strains

For ets21c experiments, a piggybac allele (Bloomington 18678) was compared to its parental strain, white^1118^ (Bloomington 6326) and an RNAi fly line (Vienna 106153) was crossed with GAL4 driver lines to elicit knockdown of ets21c. For wntD experiments, the knock out strain WntDKO1 was compared to its parental line yw and an RNAi fly line (Vienna 15146) was crossed to GAL4 driver lines to elicit knockdown of the WntD. [20] For RNAi experiments the following GAL4 driver lines from Bloomington were used: collagen25c (7011), mef-2 (27390), daughterless (8641), act5c (4414), elav (8765), lsp2 (6357), hemese (8700), and hemolectin (6395), appl (32040). RNAi experiments were conducted by crossing virgins from the RNAi line to males from driver line and collecting the progeny. If driver lines or RNAi lines contained a balancer, the progeny without the balancer were selected. Two control crosses were used for each RNAi experiment; an RNAi control with RNAi line virgins crossed to w^1118^ and a driver control with w^1118^ virgins crossed to males from the driver line.

### Bacterial strains and culture conditions

*Listeria monocytogenes* (strain 10403S) cultures were grown in 4 ml brain heart infusion (BHI) broth at 37°C without shaking after inoculation from *L. monocytogenes* grown overnight on a Luria Bertani (LB) agar plate. *L. monocytogenes* stocks were stored at −80°C in BHI broth containing 15% glycerol.

*Streptococcus pneumonia*e (strain SP1) cultures were grown standing at 37°C 5% CO_2_ in BHI broth to an OD_600_ of 0.15, and aliquots were frozen at −80°C in 10% glycerol. For infection, an aliquot of *S. pneumoniae* was thawed, diluted 1∶3 in fresh BHI broth and allowed to grow at 37°C 5% CO_2_ for 3–4 hours.

*Escherichia coli* (strain DH5a) cultures were grown in 4 ml LB broth at 37°C with shaking after inoculation from bacteria grown overnight on a Luria Bertani (LB) agar plate. *E. coli* stocks were stored at −80°C in BHI broth containing 15% glycerol.

For infection, 50 nL of the bacterial cultures were injected at the following optical densities (OD_600_): *L. monocytogenes*, 0.01 (approx. 1,000 CFU/fly); *S. pneumoniae*, 0.05–0.3 (approx 2,000–10,000 CFU/fly); *E. coli*, 0.1 (approx. 3,000 CFU/fly).

### Injection

Five to seven day post-eclosion male flies were used for injection. The flies were raised at 25°C, 65% humidity on yeasted dextrose food in a light cycling incubator (12 hours dark, 12 hours light). Flies were anesthetized with CO_2_. A picospritzer (Parker Hannin, http://www.parker.com) was used to inject 50 nL of liquid into each fly with pulled glass capillary needles that were individually calibrated by measuring the size of the expelled drop under oil. About 20 flies were placed per vial and then experiments were kept at 29°C, 65% humidity in a light cycling incubator.

### Survival experiments

Mutant flies and the parental control or RNAi crosses and RNAi/Driver controls were injected with 50 nL of the bacterial culture or medium. About sixty flies were assayed for each condition and placed in three vials of 20 flies each. Death was recorded daily. Survival curves are plotted as Kaplan-Meier plots and statistical significance is tested using log-rank analysis using Prism software (http://www.prism-software.com). All experiments were performed at least three times and yielded similar results.

### CFU determination

Colony forming units (CFUs) were determined using both spot-plating and an autoplate spiral plater (Spiral Biotech http://www.aicompanies.com). For spot-plating, eight individual flies were collected at each time point. These flies were homogenized, diluted serially and plated onto the appropriate media (blood agar for *S. pneumoniae* and LB agar for *L. monocytogenes* and *E. coli*) and grown overnight at 37°C (5% CO_2_ for *S. pneumoniae*). Some *L. monocytogenes* experiments were completed using the Spiral Biotech plater and for these six individual flies were homogenized and diluted. 50 µL of liquid was plated exponentially on a LB plate, grown overnight at 37°C and then counted using QCount, which back calculates the original number of CFU per fly. For statistical analysis, if CFU/fly did not approximate a Gaussian distribution we analyzed the log(10) transform of the data. Most CFU experiments were assessed for sources of variation using a two-way ANOVA and followed with Bonferroni post-tests for specific comparisons of interest.

Gentamicin chase assays were performed as described by Ayres et al. 2008. [4] For the zero hour time point, flies were pre-injected with 50 nL of water or gentamicin. Flies were then injected with 50 nL of L. monocytogenes and put at 29°C. The flies were incubated for three hours and then plated to determine CFUs as described above. For 24 and 48 hour time points, flies were injected with 50 nL of water or gentamicin, incubated for three hours at 29°C and similarly plated. The statistical significance of specific comparisons of interest was assessed using a two-tailed t-test.

### Microarray experiment

Flies with either injected with 50 nL of *L. monocytogenes* or BHI broth, simply stabbed with an empty needle or left unmanipulated. They were placed at 29°C for 6 hours. Groups of 20 flies were flash frozen in a dry ice/ethanol bath and then homogenized in TriZOL. Additional flies were injected and monitored for survival and CFUs to ensure adequate infection. RNA was isolated using a standard TriZOL preparation and then labeled cDNA was generated and hybridized to the Genome Drosophila Array (2.0) as described in the Affymetrix protocol (Affymetrix, http://www.affymetrix.com). Gene lists were assembled using comparisons done with dCHIP (http://biosun1.harvard.edu/complab/dchip). Anti-microbial peptide heatmap (Figure S3) created in Genespring 12.0. Select genes were confirmed by qRT-pCR.

### Quantitative RT-PCR

Flies were injected with 50 nL of the indicated microbes or kept unmanipulated. Following injection, the flies were placed in dextrose vials and incubated at 29°C for six hours. Groups of 12 flies were homogenized in TriZOL and stored at −80°C until processed. RNA was isolated using a standard TriZOL preparation, and the samples were treated with DNase (Promega, http://www.promega.com). Quantitative RT-PCR was performed as described previously by Schneider et al. using a Bio-Rad icycler and the following primer sets: WntD, Diptericin, Cecropin, and RpS15Aa (for primer sequences see Table S1) [26].

### Phagocytosis assay

These assays were performed as described by Shirasu-Hiza et al [6]. Briefly, flies were injected with 50 nL of 1 mg/ml pHrodo labeled E. coli (Molecular Probes, cat# P35361) and allowed to phagocytose at room temperature for 30–60 minutes. The wings of the flies were removed and the flies pinned onto a silicon pad with a minutien pin. Fluorescent images were taken of the dorsal surface using epifluorescent illumindation with Leica MZ3 microscope fitted with an ORCA camera. Images were captured with Openlab (Improvision), and exposures were set so that the brightest images showed no saturated pixels. Each experiment was repeated three times with 6–12 flies with similar results.

### Melanization assay

These assays were performed as described by Ayres et al [2]. Briefly, four days after injection flies were visualized by light microscopy and scored for a disseminated melanization response. Flies that melanized beyond the initial site of injection were scored positive for melanization response. Flies that only melanized at the site of injection were scored as negative for a melanization response.

## Supporting Information

Dataset S1 **Complete microarray dataset.** Includes details of the complete microarray results and analysis. Results are summarized in tables listing the top genes significantly changed during mutant-parental comparisons.
(DOCX)

Figure S1 **WntD phenotypes confirmed by RNAi knockdown.** *L. monocytogenes* or *S. pneumoniae* were injected into RNAi crosses and control flies. Survival and growth of the bacteria was monitored over the course of infection. (A),(C) L. monocytogenes; (B),(D) S. pneumoniae. Log-rank analysis of the survival curves give p<0.0001 for all curves (w/o media controls in analysis). The significant sources of variation were assessed by two-way ANOVA and differences in bacterial load between the driven RNAi and the controls at each time point were assessed by the Bonferroni post-test after ANOVA and significantly different values denoted by asterisk (* p<0.05, ** p<0.01, *** p<0.001).
(TIF)

Figure S2 **Drivers for a variety of tissues yield** ***L. monocytogenes*** **susceptibility.** *L. monocytogenes* (OD_600_ = 0.01) or media was injected into RNAi crosses and control flies. Survival was monitored over the course of infection. (A) act5c-gal4 driver, (B) da-gal4 driver, (C) hml-gal4 driver, (D) he-gal4 driver, (E) mef2-gal4 driver, (F) elav-gal4 driver. Log-rank analysis of the survival curves give p<0.0001 for all curves (w/o media control in analysis).
(TIF)

Figure S3 **Antimicrobial peptides are induced similarly in ets21c mutants.** Heatmap generated by Genespring 12.0 with a custom list of anti-microbial peptide genes. Fold changes range from −4.6 (deepest blue) to 4.6 (deepest red).
(TIF)

Table S1 **qRT-PCR primer sequences.** Sequences of both the forward and reverse primers used for the qRT-PCR experiments.
(DOCX)
