# Supplementary material for: How the Fly Balances Its Ability to Combat Different Pathogens
Source: PLoS Pathog. 2012 Dec 13;8(12):e1002970. doi: 10.1371/journal.ppat.1002970 (PMC3521699; doi:10.1371/journal.ppat.1002970)
Supplement: Dataset S1 — Complete microarray dataset. Includes details of the complete microarray results and analysis. Results are summarized in tables listing the top genes significantly changed during mutant-parental comparisons. (DOCX) [file ppat.1002970.s001.docx]

### *L. monocytogenes* has a broad effect on transcription in *Drosophila melanogaster*

While previous microarrays have been done in adult flies with *L. monocytogenes*, this is the first microarray performed with this parental strain, w1118. As parental lines respond differently to infection, looking at genes changing in this line were necessary to adequately compare changes caused by an interruption in ets21c.

We looked at parental line changes in two contexts. First, we looked for any gene that changed during *L. monocytogenes* infection compared to unmanipulated flies, including genes induced through wounding and media injection. Second, we specifically selected for genes which changed during *L. monocytogenes* infection, but not during wounding. Both of these classes are important for the immune response to and eventual survival during infection, but considering them separately allows for proper interpretation.

Looking at all changes during infection, 232 genes were significantly different between unmanipulated and *L. monocytogenes* infected flies (p<0.01). Gene Ontology (GO) analysis revealed many expected categories including humoral immune response, defense response, and the Toll signaling pathway. There was also enrichment in a number of metabolic categories: carbohydrate metabolism, electron transport, hexose metabolism, lipid metabolism, monosaccharide metabolism, pigment metabolism, protein metabolism, and secondary metabolism. A list of the genes with changes greater than 4.5 fold is in Supplemental Table 2.

After filtering out genes affected by wounding, we found *L. monocytogenes* infection caused changes in 146 genes listed in Supplemental Table 3. This list was generated by selecting genes which significantly changed between unmanipulated and *L. monocytogenes* infected flies (p<0.01) and removing any genes which were significantly different between unmanipulated and media injected flies (p<0.1). Gene Ontology analysis revealed similar results to those above, expected immune clusters – humoral immune response, defense response, and Toll signaling pathway – and metabolic clusters – carbohydrate metabolism, electron transport, lipid metabolism and pigment metabolism.

A closer look at regulated immunity genes reveals that Toll signaling genes are up-regulated 2-4 fold during *L. monocytogenes* infection. These genes are not significantly induced by media injection, but the downstream read-outs of the pathways, anti-microbial peptides, are. The induction of these genes indicates activation of both the imd and the Toll pathways. Gregorio et al showed that the Toll components that we see up-regulated (cactus, pelle, Toll, spaetzle, and necrotic) as well as additional factors like relish and frost are all spaetzle signaling dependent.[[1](#_ENREF_1)] Of the anti-microbial peptides significantly induced, attacin-A, attacin-B, drosomycin, and metchnikowan can be induced by either Toll or imd; attacin-C and diptercin B require imd signaling; and defensin requires signaling through both toll and imd pathways.[[1](#_ENREF_1)] *L. monocytogenes* is gram-positive bacterium that excites both the Toll and imd pathways and yet *L. monocytogenes* possesses a DAP-type peptidoglycan which, according to *Drosophila* dogma, should only cause signaling through the imd pathway.

In the genes significantly down-regulated during *L. monocytogenes* infection, there is significant enrichment in metabolic pathways. Transcripts in the glycolysis/gluconeogenisis, beta-oxidation, mitochondrial fatty acid metabolism and the pentose-phosphate pathway are depressed during infection. This could be indicative either of loss of substrates or of a regulatory shift on the fly’s part in an effort to regulate energy usage.

Miscellaneous other genes of interest include frost, which affects cold resistance and is transcriptioanlly induced during other infections, and adipokinetic hormone receptor, which is important for triglyceride utilization.[[1-3](#_ENREF_1)]

### Et21c mutant flies have few baseline significant differences from parental

To select for genes that are different in ets21c mutants in the absence of infection we compared unmanipulated parental to unmanipulated ets21c mutants (Supplementary Table 4). There were 34 genes which had significantly different basal expression levels between the ets21c mutant and parental line (p<0.01), and this gene list was enriched for genes in the defense response: lysozyme X, drosomycin-4, immune induced molecule 23, ebony, Ugt86dD, CG31832, and CG16799.

### Ets21c mutant flies induce fewer genes during *L. monocytogenes* infection

*L. monocytogenes* infection of ets21c piggybac mutants caused significant changes in 41 genes (p<0.01) and only 18 of these genes do not change during media injection as compared to the 236 and 141 genes changes under the same conditions, respectively, in the parental line (Supplementary Table 5). Gene Ontology analysis revealed enrichment in humoral immune response, defense response and toll signaling pathway. Notably missing, compared to the parental results, was enrichment in the metabolic systems. There was enrichment in carbohydrate catabolism, but this is a distinct GO group from those seen in the parental line.

To look at which genes are induced during parental infection but not during ets21c mutant infections, we took the list of genes significantly changed between unmanipulated and *L. monocytogenes* infected in the parental line (p<0.01), selected against genes changed through media injection (p<0.1), and selected against genes changed in ets21c mutants (p<0.1). We used higher p-values for our anti-selection criteria to have the strictest criteria for gene selection.

The anti-selection of both media injection and ets21c was mostly redundant, with the ets21c selection criteria only removing two additional genes. To loosen selection criteria, we looked at all genes which change in the parental line during *L. monocytogenes* infection (unmanipulated versus *L. monocytogenes*, p<0.01) and filtered for genes which changed during ets21c piggybac infection (unmanipulated vs. *L monocytogenes*, p<0.1). This criterion eliminated 41 genes, leaving a list of 191 genes which were not affected by infection in the ets21c mutant (Supplementary Table 2). These genes almost entirely coincide with genes affected by media injection, hence the low level of additional selection by introducing both restrictions.

The gene most highly induced during *L. monocytogenes* infection in the parental line, but not in the ets21c mutant and during wounding, was wntD. Mutants in wntD are susceptible to *L. monocytogenes* infection, making this a potential mediator of ets21c’s immune phenotype.

One of the genes most down-regulated during infection in the parental line, but not affected in the ets21c mutant, is the gene adipokinetic hormone receptor (akhr). Akhr is the *Drosophila* equivalent of the mammalian glucagon receptor and is responsible to controlling the release of triglyceride stores.[[2](#_ENREF_2),[3](#_ENREF_3)]

### Ets21c mutants have a different expression signature during *L. monocytogenes* infection

Because there are so few changes induced in the ets21c mutants by *L. monocytogenes* infection, we also looked at a direct comparison between *L. monocytogenes* infected ets21c mutant versus infected parental. When we did this comparison, 124 genes were significantly different (p<0.01) (Supplementary Table 6). This revealed both genes induced during infection and baseline differences, when we selected against the baseline differences in unmanipulated flies (p<0.1) we reduced the number to 94 genes. GO analysis of the non-baseline differences were enriched in catabolism, electron transport, iron binding, oxidoreductase activity, and purine nucleotide metabolism. This selection condition again highlights WntD and akhr as potential mediators of the ets21c phenotype. This criterion also highlights the gene frost, which is strongly induced in the parental line during infection (~20 fold) but is only induced four-fold in the ets21c mutant. As mentioned above, frost affects cold tolerance in *Drosophila*, and is known to be immune induced by a number of microbes.[[4-6](#_ENREF_4)]

Supplemental Table 2. Genes regulated during *L. monocytogenes* infection in parental line.

| Gene Name | CG | Fold Change (Unmanip vs. Listeria) | Significant change during media injection? | Significant change in ets21c mutant during infection? |
| --- | --- | --- | --- | --- |
| CG4950 | CG4950 | -15.68 | * | * |
| CG16756 | CG16756 | -15.12 | * |  |
| female-specific independent of transformer | CG17820 | -14.2 | * |  |
| Cytochrome b5-related | CG13279 | -13.67 | * |  |
| Adipokinetic hormone receptor | CG11325 | -12.06 / -2.98 | */ |  |
| Cyp4ac2 | CG17970 | -10.5 | * |  |
| CG6067 | CG6067 | -10.04 |  |  |
| CG31778 | CG31778 | -9.53 | * |  |
| Lipid storage droplet-1 | CG10374 | -7.92 | * |  |
| CG8147 | CG8147 | -7.91 | * |  |
| CG2736 | CG2736 | -7.79 | * |  |
| Retinoid- and fatty-acid binding protein | CG11064 | -7.05 | * |  |
| CG4716 | CG4716 | -6.74 | * |  |
| Glutactin | CG9280 | -6.44 |  |  |
| CG9466 | CG9466 | -6.32 | * |  |
| CG5428 | CG5428 | -6.29 | * |  |
| lectin-33A | CG16834 | -6.15 | * | * |
| CG15092 | CG15092 | -6.04 | * |  |
| nicotinic acetylcholine receptor beta 21C | CG11822 | -5.71 | * |  |
| Hemolectin | CG7002 | -5.59 |  |  |
| Papilin | CG33103 | -5.54 | * | * |
| CG3597 | CG3597 | -5.47 | * |  |
| CG10131 | CG10131 | -5.36 |  |  |
| Phosphoribosylamidotransferase 2 | CG10078 | -5.2 |  |  |
| CG14990 | CG14990 | -5.16 | * |  |
| CG4716 | CG4716 | -5.16 |  |  |
| CG9468 | CG9468 | -5.14 | * |  |
| CG13877 | CG13877 | -5.11 |  |  |
| CG13607 | CG13607 | -5 |  |  |
| regucalcin | HDC18536 | -4.99 / -2.59 | */ | * |
| CG9837 | CG9837 | -4.87 | * | * |
| Sorbitol dehydrogenase 1 | CG1982 | -4.68 | * |  |
| CG6503 | CG6503 | -4.64 |  |  |
| CG18249 | CG18249 | -4.61 | * |  |
| CG5288 | CG5288 | -4.58 |  |  |
| Serine pyruvate aminotransferase | CG3926 | -4.56 |  |  |
| CG15199 | CG15199 | -4.52 |  |  |
| PGRP-SB1 | CG9681 | 4.57 | * | * |
| Activin Like Protein at 23B | CG16987 | 4.6 |  |  |
| Attacin-C | CG4740 | 4.62 | * |  |

#### Supplemental Table 2. Genes regulated during *L. monocytogenes* infection in parental line (continued).

| Gene Name | CG Number | Fold Change | Significant change during media injection? | Significant change in ets21c mutant during infection? |
| --- | --- | --- | --- | --- |
| PGRP-SB1 | CG9681 | 4.57 | * | * |
| Activin Like Protein at 23B | CG16987 | 4.6 |  |  |
| Attacin-C | CG4740 | 4.62 | * |  |
| CG9542 | CG9542 | 4.64 |  |  |
| CG1299 | CG1299 | 4.67 |  |  |
| Ser7 | CG2045 | 4.83 | * |  |
| CG14205 | CG14205 | 5.09 |  |  |
| PGRP-SC2 | CG14745 | 5.16 | * | * |
| CG1102 | CG1102 | 5.22 |  |  |
| Immune induced molecule 1 | CG18108 | 5.33 | * |  |
| CG6639 | CG6639 | 5.49 | * |  |
| CG4267 | CG4267 | 5.73 | * | * |
| CG32373 | CG32373 | 5.74 | * |  |
| Thiolester containing protein I | CG18096 | 5.77 | * | * |
| CG9080 | CG9080 | 5.85 | * | * |
| Immune induced molecule 10 | CG18279 | 6.01 | * | * |
| DiptericinB | CG10794 | 6.31 | * | * |
| CG2444 | CG2444 | 6.54 |  |  |
| CG6361 | CG6361 | 6.6 | * | * |
| CG5527 | CG5527 | 6.63 | * | * |
| Attacin-B | CG18372 | 6.79 | * | * |
| CG6553 | CG6553 | 6.88 | * | * |
| CG8791 | CG8791 | 7.2 |  |  |
| CG11413 | CG11413 | 7.87 | * | * |
| PGRP-SD | CG7496 | 8.35 | * | * |
| CG6687 | CG6687 | 8.71 | * | * |
| Attacin-A | CG10146 | 8.99 | * | * |
| CG9989 | CG9989 | 9.13 | * | * |
| CG14516 | CG14516 | 9.56 |  |  |
| yellow-f | CG18550 | 11.46 | * | * |
| CG18563 | CG18563 | 11.85 | * | * |
| CG33307 | CG16887 | 12.52 | * |  |
| wnt inhibitor of Dorsal | CG8458 | 17.37 |  |  |
| CG2217 | CG2217 | 17.38 | * | * |
| CG30080 | CT32157 | 17.86 | * | * |
| CG30080 | CG30080 | 18.63 | * | * |
| Frost | CG9434 | 23.21 | * | * |
| suppressor of white-apricot | CG3019 | 26.48 | * |  |
| CG14567 | CG14567 | 33.71 | * |  |
| Immune induced molecule 23 | CG15066 | 37.39 | * | * |
| CG11459 | CG11459 | 37.56 | * | * |
| CG6675 | CG6675 | 42.41 | * | * |
| CG31775 | CG31775 | 70.51 | * | * |

Supplemental Table 3. Genes regulated by *L. monocytogenes* infection but not wounding.

| **Gene Name** | **CG**  **Number** | **Fold Change** |  | **Gene Name** | **CG**  **Number** | | **Fold Change** |
| --- | --- | --- | --- | --- | --- | --- | --- |
| CG6067 | CG6067 | -10.04 |  | wnt inhibitor of Dorsal | | CG8458 | 17.37 |
| CG2736 | CG2736 | -7.79 |  | CG14516 | | CG14516 | 9.56 |
| CG4716 | CG4716 | -6.74 |  | CG8791 | | CG8791 | 7.2 |
| Glutactin | CG9280 | -6.44 |  | CG2444 | | CG2444 | 6.54 |
| Hemolectin | CG7002 | -5.59 |  | CG1102 | | CG1102 | 5.22 |
| CG10131 | CG10131 | -5.36 |  | CG14205 | | CG14205 | 5.09 |
| Phosphoribosylamido-transferase 2 | CG10078 | -5.2 |  | CG1299 | | CG1299 | 4.67 |
| CG4716 | CG4716 | -5.16 |  | CG9542 | | CG9542 | 4.64 |
| CG13877 | CG13877 | -5.11 |  | Activin Like Protein at 23B | | CG16987 | 4.6 |
| CG13607 | CG13607 | -5 |  | CG1698 | | CG1698 | 4.42 |
| CG6503 | CG6503 | -4.64 |  | CG4618 | | CG4618 | 4.39 |
| CG5288 | CG5288 | -4.58 |  | CG17350 | | CG17350 | 4.26 |
| Serine pyruvate aminotransferase | CG3926 | -4.56 |  | CG31617 | | CG31617 | 4.26 |
| CG15199 | CG15199 | -4.52 |  | cactus | | CG5848 | 4.15 |
| CG14400 | CG14400 | -4.33 |  | CG11843 | | CG11843 | 3.98 |
| lectin-24Db | CG2958 | -4.33 |  | CG32412 | | CG32412 | 3.94 |
| CG10361 | CG10361 | -4.21 |  | CG18530 | | CG18530 | 3.88 |
| Cytochrome P450-18a1 | CG6816 | -4.16 |  | Defensin | | CG1385 | 3.84 |
| Ance-4 | CG8196 | -4.07 |  | CG3505 | | CG3505 | 3.79 |
| CG2801 | CG2081 | -4.03 |  | p115 | | CG1422 | 3.75 |
| Open rectifier K<up>+</up> channel 1 | CG1615 | -3.99 |  | Relish | | CG11992 | 3.73 |
| CG9295 | CG9295 | -3.97 |  | lozenge | | CG1689 | 3.72 |
| ade5 | CG3989 | -3.89 |  | Serpin-27A | | CG11331 | 3.71 |
| CG10621 | CG10621 | -3.79 |  | Dopa decarboxylase | | CG10697 | 3.67 |
| CG13082 | CG13082 | -3.74 |  | CG7219 | | CG7219 | 3.62 |
| sex-specific enzyme 2 | CG4979 | -3.65 |  | CG13075 | | CG13075 | 3.56 |
| CG32072 | CG32072 | -3.63 |  | spatzle | | CG6134 | 3.55 |
| CG5958 | CG5958 | -3.62 |  | CG17119 | | CG17119 | 3.49 |
| Collagen type IV | CG4145 | -3.53 |  | Spn43Ad | | CG1859 | 3.45 |
| Cyp28d1 | CG10833 | -3.52 |  | pitchoune | | CG6375 | 3.4 |
| CG33047 | CG33047 | -3.49 |  | CG34037 | | CG34037 | 3.39 |
| CG6034 | CG6034 | -3.45 |  | Suppressor of cytokine signaling at 36E | | CG15154 | 3.23 |
| Cyp6t1* | CG1644 | -3.45 |  | CG6388 | | CG6388 | 3.21 |
| Iris | CG4715 | -3.42 |  | CG8913 | | CG8913 | 3.21 |
| CG9497* | CG9497 | -3.25 |  | nop5 | | CG10206 | 3.12 |
| Glycerol 3 phosphate dehydrogenase | CG9042 | -3.25 |  | CG5773 | | CG5773 | 3.04 |
| Cytochrome P450-4d1 | CG3656 | -3.24 |  | CG4927 | | CG4927 | 3.02 |
| CG33493 | CG33493 | -3.1 |  | Pherokine 3 | | CG9358 | 2.99 |
| Ser8 | CG4812 | -3.07 |  | CG1607 | | CG1607 | 2.88 |
| CG11407 | CG11407 | -3.06 |  | CG5246 | | CG5246 | 2.88 |
| CG14523 | CG14523 | -3.05 |  | PDGF- and VEGF-related factor 2 | | CG13780 | 2.88 |

#### Supplemental Table 3. Genes regulated by *L. monocytogenes* infection but not wounding (continued).

| **Gene Name** | **CG Number** | **Fold Change** |  | **Gene Name** | **CG Number** | **Fold Change** |
| --- | --- | --- | --- | --- | --- | --- |
| Imaginal disc growth factor 5 | CG5154 | -3.04 |  | Punch | CG9441 | 2.87 |
| Adipokinetic Hormone receptor | CG11325 | -2.98 |  | CG10627 | CG10627 | 2.83 |
| CG3523 | CG3523 | -2.97 |  | CG11034 | CG11034 | 2.81 |
| Esterase-7 | CG1112 | -2.92 |  | CG12772 | CG12772 | 2.74 |
| CG11892 | CG11892 | -2.88 |  | CG9616 | CG9616 | 2.74 |
| CG9511 | CG9511 | -2.86 |  | Urate oxidase | CG7171 | 2.7 |
| CG32158 | CG32158 | -2.82 |  | Cyclic-AMP response element binding protein A | CG7450 | 2.68 |
| Trypsin | CG12385 | -2.82 |  | pelle | CG5974 | 2.63 |
| acyl-Coenzyme A oxidase at 57D distal | CG9709 | -2.81 |  | Serine protease inhibitor 5 | CG18525 | 2.62 |
| CG15203 | CG15203 | -2.8 |  | CG31686 | CG31686 | 2.61 |
| CG4019 | CG4019 | -2.8 |  | rdgB | CG17818 | 2.59 |
| pugilist | CG4067 | -2.8 |  | CG12116 | CG12116 | 2.57 |
| CG16926 | CG16926 | -2.77 |  | nervy | CG3385 | 2.57 |
| CG14375 | CG14375 | -2.76 |  | CG15044 | CG15044 | 2.56 |
| CG15096 | CG15096 | -2.74 |  | CG9809 | CG9809 | 2.56 |
| CG33120 | CG33120 | -2.73 |  | Arginine methyltransferase 8 | CG16840 | 2.54 |
| Cyp9h1 | CG17577 | -2.72 |  | CDC45L | CG3658 | 2.51 |
| CG18301 | CG18301 | -2.69 |  | CG31326 | CG31326 | 2.48 |
| Rhythmically expressed gene 2 | CG3200 | -2.69 |  | CG7115 | CG7115 | 2.46 |
| Trehalose-6-phosphate synthase 1 | CG4104 | -2.69 |  | CG3781 | CG3781 | 2.45 |
| CG15406 | CG15406 | -2.64 |  | CG5550 | CG5550 | 2.42 |
| fructose-1,6-bisphosphatase | CG31692 | -2.64 |  | CG18348 | CG18348 | 2.41 |
| CG14823 | CG14823 | -2.6 |  | Misexpression suppressor of KSR 4 | CG31447 | 2.4 |
| CG7322 | CG7322 | -2.59 |  | CG33258 | CR33258 | 2.36 |
| CG6206 | CG6206 | -2.56 |  | CG9416 | CG9416 | 2.35 |
| CG8654 | CG8654 | -2.54 |  | CG15043 | CG15043 | 2.19 |
| CG9510 | CG33085 | -2.53 |  |  |  |  |
| Olfactory-specific 9 | CG10658 | -2.53 |  |  |  |  |
| CG11315 | CG11315 | -2.49 |  |  |  |  |
| Senescence marker protein-30 | CG7390 | -2.47 |  |  |  |  |
| real-time | CG9528 | -2.46 |  |  |  |  |
| CG5560 | CG5560 | -2.39 |  |  |  |  |
| CG11314 | CG11314 | -2.37 |  |  |  |  |
| CG32667 | CG32667 | -2.34 |  |  |  |  |
| Cyp6g1 | CG8453 | -2.3 |  |  |  |  |
| Odorant-binding protein 99c | CG7584 | -2.28 |  |  |  |  |

Supplemental Table 4. Genes that are differentially expressed at baseline between ets21c mutants and the parental line.

| **Gene Name** | **CG number** | **Fold Change** |
| --- | --- | --- |
| CG5139 | CG5139-RA | -33.21 |
| Cyp6a17 | CG10241-RA | -28.9 |
| CG18066 | CG18066-RA | -14 |
| CG5381 | CG5381-RA | -11.23 |
| Larval serum protein 2 | CG6806-RA | -7.4 |
| CG31832 | CG31832-RA | -6.91 |
| CG4409 | CG4409-RA | -5.87 |
| Lysozyme X | CG9120-RA | -4.77 |
| Larval serum protein 1α | CG2559-RA | -4.17 |
| insulin-like peptide 5 | HDC09365 | -4.06 |
| CG14115 | CG14115-RA | -3.56 |
| CG31672 | CG31672-RA | -3.4 |
| Cyp9h1 | CG17577-RA | -3.07 |
| CG16704 | CG16704-RA | -3.04 |
| CG14499 | CG14499-RA | -2.63 |
| ebony | CG3331-RA | -2.33 |
| Larval serum protein 1β | CG4178-RA | -2.26 |
| CG16799 | CG16799-RA | -2.25 |
| CG11294 | CG11294-RA | 2.56 |
| CG11893 | CG11893-RA | 2.58 |
| Cyp6a23 | CG10242-RA | 2.8 |
| Insulin-related peptide | CG8167-RA | 2.87 |
| drosomycin-4 | CG32282-RA | 3.03 |
| CG10550 | CG10550-RB | 3.24 |
| CG3397 | CG3397-RA | 3.62 |
| Immune induced molecule 23 | CG15066-RA | 4.15 |
| CG11909 | CG11909-RA | 4.69 |
| CG32036 | CG32036-RB | 4.94 |
| CG7900 | CG7900-RA | 5.06 |
| Ugt86Dd | CG6633-RA | 5.29 |
| Syt7 | CG2381-RB | 6.15 |
| Esterase-2 | CG2505-RA | 6.55 |
| CG13313 | CG13313-RA | 6.89 |
| CG13155 | CG13155-RA | 75.97 |

Supplemental Table 5. Genes regulated during *L. monocytogenes* infection in ets21c mutants.

| **Gene Name** | **Accession** | **Fold Change** | **Significant change during media injection?** |
| --- | --- | --- | --- |
| CG4950 | CG4950-RA | -6.63 | * |
| regucalcin | HDC18536 | -3.43 | * |
| neither inactivation nor afterpotential D | CG31783-RA | -2.74 |  |
| CG34020 | Dm.2R.20325.0 | -2.53 |  |
| CG12374 | CG12374-RA | -2.47 |  |
| CG11594 | CG11594-RC | -2.36 |  |
| CG9631 | CG9631-RA | 2.26 |  |
| Relish | CG11992-RA | 2.34 |  |
| CG15021 | CG15021-RA | 2.47 |  |
| Cyp309a1 | CG9964-RA | 2.53 |  |
| CG17325 | CG17325-RA | 2.54 |  |
| Mec2 | CG7635-RA | 2.57 |  |
| Diptericin B | CG10794-RA | 2.73 | * |
| CG13422 | CG13422-RA | 2.8 | * |
| Immune induced molecule 10 | CG33470 | 2.83 | * |
| Attacin-B | CG18372-RA | 2.88 | * |
| CG3505 | CG3505-RA | 2.89 |  |
| NAD-dependent methylenetetrahydrofolate dehydrogenase | CG18466-RB | 2.9 |  |
| CG17032 | CG17032-RA | 2.91 |  |
| CG6553 | CG6553-RA | 3.13 | * |
| CG5527 | CG5527-RA | 3.2 | * |
| Thiolester containing protein II | CG7052-RA | 3.24 | * |
| CG6385 | CG6385-RA | 3.27 | * |
| CG6687 | CG6687-RA | 3.41 |  |
| PGRP-SC2 | CG14745-RA | 3.46 |  |
| Immune induced molecule 10 | CG18279-RB | 3.69 | * |
| CG11413 | CG11413-RA | 3.74 |  |
| CG30080 | CT32157 | 3.94 |  |
| CG31495 | CG31495-RA | 3.97 |  |
| CG30080 | CG30080-RA | 3.99 |  |
| Peptidoglycan recognition protein SA | CG11709-RA | 4.14 | * |
| yellow-f | CG18550-RA | 4.22 | * |
| CG4267 | CG4267-RA | 4.62 | * |
| PGRP-SB1 | CG9681-RA | 4.94 | * |
| CG32185 | CG32185-RA | 5 | * |
| CG6361 | CG6361-RA | 5.36 | * |
| CG13077 | CG13077-RA | 5.66 | * |
| Immune induced molecule 23 | CG15066-RA | 5.8 | * |
| PGRP-SD | CG7496-RA | 6.97 | * |
| CG2217 | CG2217-RA | 7.22 | * |
| CG9989 | CG9989-RA | 8.94 | * |

Supplemental Table 6. Genes with differential expression between ets21c mutants and parental during *L. monocytogenes* infection.

| **Gene Name** | **CG Number** | **Fold Change** | **Changed in baseline?** |
| --- | --- | --- | --- |
| CG5139 | CG5139-RA | -37.73 | * |
| CG14934 | CG14934-RA | -29.01 | * |
| Cyp6a17 | CG10241-RA | -26.62 | * |
| CG18066 | CG18066-RA | -25.25 | * |
| wnt inhibitor of Dorsal | CG8458-RA | -21.75 |  |
| CG2444 | CG2444-RA | -11.8 |  |
| CG5381 | CG5381-RA | -11.68 | * |
| CG6675 | CG6675-RA | -9.04 |  |
| CG14567 | CG14567-RA | -6.79 |  |
| CG31832 | CG31832-RA | -6.49 | * |
| CG8791 | CG8791-RA | -6.46 |  |
| CG14516 | CG14516-RB | -6.31 |  |
| Larval serum protein 2 | CG6806-RA | -6.3 | * |
| CG33462 | CG30086-RA | -5.58 |  |
| CG4409 | CG4409-RA | -5.09 | * |
| Frost | CG9434-RA | -5.08 |  |
| CG33307 | CG16887-RA | -4.85 |  |
| suppressor of white-apricot | CG3019-RA | -4.85 | * |
| GCR(ich) | CG5812-RA | -4.48 | * |
| CG18530 | CG18530-RA | -4.42 |  |
| CG9542 | CG9542-RA | -4.36 |  |
| CG4618 | CG4618-RA | -4.2 |  |
| CG16704 | CG16704-RA | -4.17 | * |
| CG11459 | CG11459-RA | -4.08 |  |
| CG17350 | CG17350-RA | -3.91 |  |
| CG13075 | CG13075-RA | -3.82 |  |
| CG32922 | CG32922-RB | -3.76 |  |
| CG18563 | CG18563-RA | -3.37 |  |
| CG17119 | CG17119-RA | -3.33 |  |
| lozenge | CG1689-RA | -3.28 |  |
| CG9416 | CG9416-RA | -3.24 |  |
| Ady43A | CG1851-RA | -3.11 |  |
| CG6553 | CG6553-RA | -2.95 |  |
| CG14205 | CG14205-RA | -2.9 |  |
| CG13325 | CG13325-RA | -2.73 |  |
| CG31672 | CG31672-RA | -2.72 | * |
| CG1102 | CG1102-RA | -2.71 |  |
| CG34037 | Dm.3R.39174.0 | -2.69 |  |
| CG1698 | CG1698-RA | -2.59 |  |
| CG6388 | CG6388-RA | -2.58 |  |
| CG32412 | CG32412-RA | -2.58 |  |
| CG4733 | CG4733-RA | -2.57 |  |
| Spn43Ad | CG1859-RA | -2.56 |  |
| CG11576 | CG11576-RA | -2.51 |  |
| CG5246 | CG5246-RA | -2.49 |  |
| yellow-f | CG18550-RA | -2.41 |  |
| CG17032 | CG17032-RA | -2.36 |  |
| CG6687 | CG6687-RA | -2.34 |  |
| spatzle | CG6134-RA | -2.32 |  |
| CG11919 /// CG18003 | CG30019-RB | 2.37 |  |

#### Supplemental Table 6. Genes with differential expression between ets21c mutants and parental during *L. monocytogenes* infection (continued).

| **Gene Name** | **CG Number** | **Fold Change** | **Changed in baseline?** |
| --- | --- | --- | --- |
| Cyp6g1 | CG8453-RA | 2.43 |  |
| Cyp4ac1 | CG14032-RA | 2.47 |  |
| CG6330 | CG6330-RB | 2.48 |  |
| Adenosine deaminase-related growth factor D | CG9621-RA | 2.52 |  |
| Phosphoribosylamidotransferase 2 | CG10078-RB | 2.57 |  |
| CG3829 | CG3829-RA | 2.61 |  |
| Insulin-related peptide | CG8167-RA | 2.64 | * |
| CG13833 | CG13833-RA | 2.66 |  |
| pugilist | CG4067-RB | 2.68 |  |
| CG11407 | CG11407-RA | 2.68 |  |
| CG6912 | CG6912-RA | 2.74 |  |
| CG10361 | CG10361-RA | 2.75 |  |
| CG15203 | CG15203-RA | 2.75 |  |
| tolkin | CG6863-RB | 2.76 |  |
| Lysozyme P | CG9116-RA | 2.83 |  |
| CG5288 | CG5288-RA | 2.83 |  |
| CG8854 | CG8854-RA | 2.87 |  |
| sex-specific enzyme 2 | CG4979-RA | 2.9 |  |
| CG34020 | Dm.2R.20325.0 | 2.91 |  |
| CG6503 | CG6503-RA | 2.99 |  |
| CG15553 | CG15553-RA | 3 |  |
| ade5 | CG3989-RA | 3.05 |  |
| CG33047 | CG33047-RA | 3.06 |  |
| CG8550 | CG8550-RA | 3.12 |  |
| CG9676 | CG9676-RA | 3.14 |  |
| CG3301 | CG3301-RA | 3.22 |  |
| Ance-4 | CG8196-RA | 3.25 |  |
| CG16732 | CG16732-RA | 3.28 | * |
| CG31380 | CG31380-RA | 3.28 |  |
| drosomycin-4 | CG32282-RA | 3.32 | * |
| CG3597 | CG3597-RA | 3.38 |  |
| Lipid storage droplet-1 | CG10374-RA | 3.49 |  |
| Cyp309a1 | CG9964-RA | 3.52 |  |
| CG4716 | CG4716-RA | 3.52 |  |
| Open rectifier K<up>+</up> channel 1 | CG1615-RB | 3.54 |  |
| CG15199 | CG15199-RA | 3.62 |  |
| Deoxyribonuclease II | CG7780-RA | 3.63 |  |
| Thd1 | HDC16707 | 3.63 | * |
| Adipokinetic Hormone receptor | CG11325-RB | 3.68 |  |
| CG13877 | CG13877-RA | 3.78 |  |
| Glutactin | CG9280-RC | 3.86 |  |
| CG14400 | CG14400-RA | 3.88 |  |
| CG5428 | CG5428-RA | 3.89 |  |
| Cyp6a23 | CG10242-RA | 3.93 | * |
| CG10550 | CG10550-RB | 3.93 | * |
| CG31778 | CG31778-RA | 3.96 |  |
| CG7542 | CG7542-RA | 4.01 | * |
| CG4716 | CG4716-RB | 4.11 |  |
| Cyp309a2 | CG18559-RA | 4.17 |  |
| CG2081-RB | CG2081-RB | 4.22 |  |

#### Supplemental Table 6. Genes with differential expression between ets21c mutants and parental during *L. monocytogenes* infection (continued).

| **Gene Name** | **CG Number** | **Fold Change** | **Changed in baseline?** |
| --- | --- | --- | --- |
| CG6415 | CG6415-RA | 4.28 |  |
| CG10131 | CG10131-RA | 4.45 |  |
| white | CG2759-RA | 4.51 | * |
| CG31091 | CG31091-RA | 4.63 |  |
| CG6067 | CG6067-RA | 4.84 |  |
| Retinoid- and fatty-acid binding protein | CG11064-RA | 5.01 |  |
| Cyp4ac2 | CG17970-RA | 5.02 |  |
| rhomboid-6 | CG17212-RB | 5.1 | * |
| CG14920 | CG14920-RA | 5.24 | * |
| CG13607 | CG13607-RA | 5.35 |  |
| CG3528 | CG3528-RA | 5.8 | * |
| CG11236 | CG11236-RA | 6.22 |  |
| CG9463 | CG9463-RA | 6.4 |  |
| CG13313 | CG13313-RA | 6.42 | * |
| Ugt86Dd | CG6633-RA | 6.99 | * |
| CG6385 | CG6385-RA | 7.07 |  |
| CG2736 | CG2736-RA | 8.54 |  |
| CG11598 | CG11598-RA | 8.81 | * |
| Cytochrome b5-related | CG13279-RA | 9.67 |  |
| CG11659 | CG11659-RA | 20.26 | * |
| CG12256 | CG12256-RA | 30.76 | * |
| CG13155 | CG13155-RA | 76.48 | * |

1. De Gregorio E, Spellman PT, Tzou P, Rubin GM, Lemaitre B (2002) The Toll and Imd pathways are the major regulators of the immune response in Drosophila. EMBO J 21: 2568-2579.

2. Bharucha KN, Tarr P, Zipursky SL (2008) A glucagon-like endocrine pathway in Drosophila modulates both lipid and carbohydrate homeostasis. J Exp Biol 211: 3103-3110.

3. Gronke S, Muller G, Hirsch J, Fellert S, Andreou A, et al. (2007) Dual lipolytic control of body fat storage and mobilization in Drosophila. PLoS Biol 5: e137.

4. Reis M, Vieira CP, Morales-Hojas R, Aguiar B, Rocha H, et al. (2011) A comparative study of the short term cold resistance response in distantly related Drosophila species: the role of regucalcin and frost. PLoS One 6: e25520.

5. Udaka H, Ueda C, Goto SG (2010) Survival rate and expression of Heat-shock protein 70 and Frost genes after temperature stress in Drosophila melanogaster lines that are selected for recovery time from temperature coma. J Insect Physiol 56: 1889-1894.

6. Colinet H, Lee SF, Hoffmann A (2010) Functional characterization of the Frost gene in Drosophila melanogaster: importance for recovery from chill coma. PLoS One 5: e10925.
