# Supplementary figures and images for: How the Fly Balances Its Ability to Combat Different Pathogens
Source: PLoS Pathog. 2012 Dec 13;8(12):e1002970. doi: 10.1371/journal.ppat.1002970 (PMC3521699; doi:10.1371/journal.ppat.1002970)

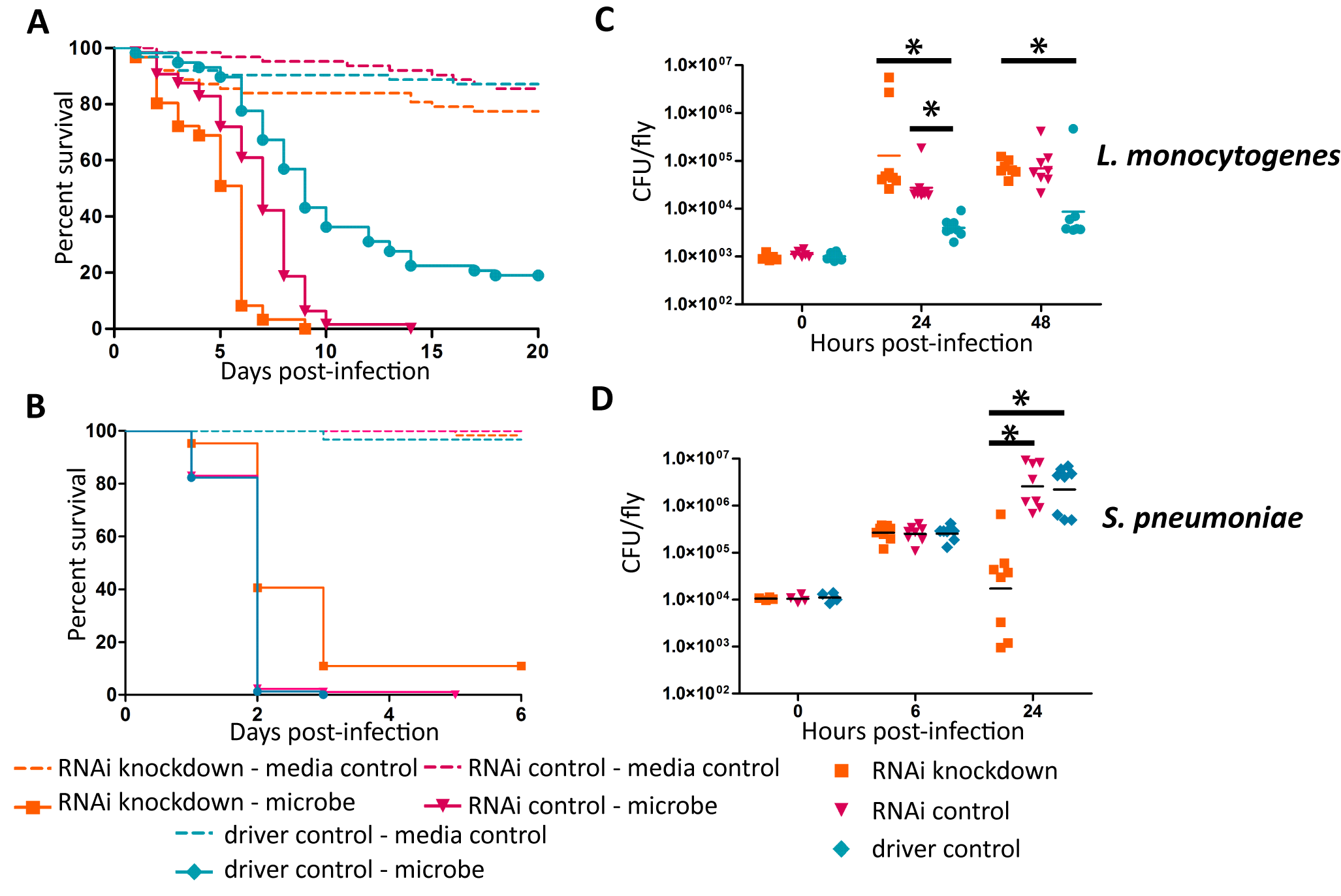

Supplement: Figure S1 — WntD phenotypes confirmed by RNAi knockdown. L. monocytogenes or S. pneumoniae were injected into RNAi crosses and control flies. Survival and growth of the bacteria was monitored over the course of infection. (A),(C) L. monocytogenes; (B),(D) S. pneumoniae. Log-rank analysis of the survival curves give p<0.0001 for all curves (w/o media controls in analysis). The significant sources of variation were assessed by two-way ANOVA and differences in bacterial load between the driven RNAi and the controls at each time point were assessed by the Bonferroni post-test after ANOVA and significantly different values denoted by asterisk (* p<0.05, ** p<0.01, *** p<0.001). (TIF) [file ppat.1002970.s002.tif]

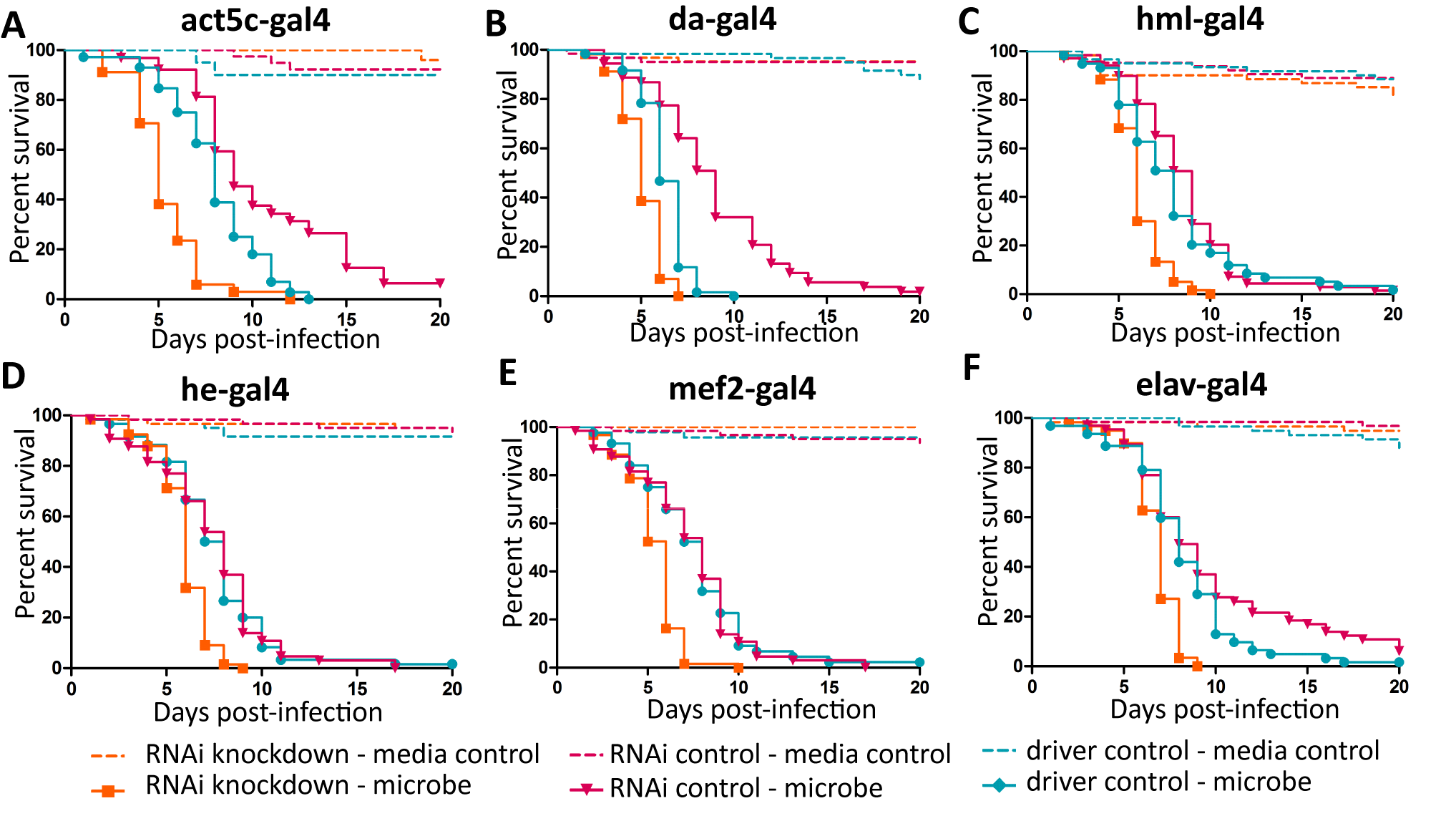

Supplement: Figure S2 — Drivers for a variety of tissues yield L. monocytogenes susceptibility. L. monocytogenes (OD600 = 0.01) or media was injected into RNAi crosses and control flies. Survival was monitored over the course of infection. (A) act5c-gal4 driver, (B) da-gal4 driver, (C) hml-gal4 driver, (D) he-gal4 driver, (E) mef2-gal4 driver, (F) elav-gal4 driver. Log-rank analysis of the survival curves give p<0.0001 for all curves (w/o media control in analysis). (TIF) [file ppat.1002970.s003.tif]

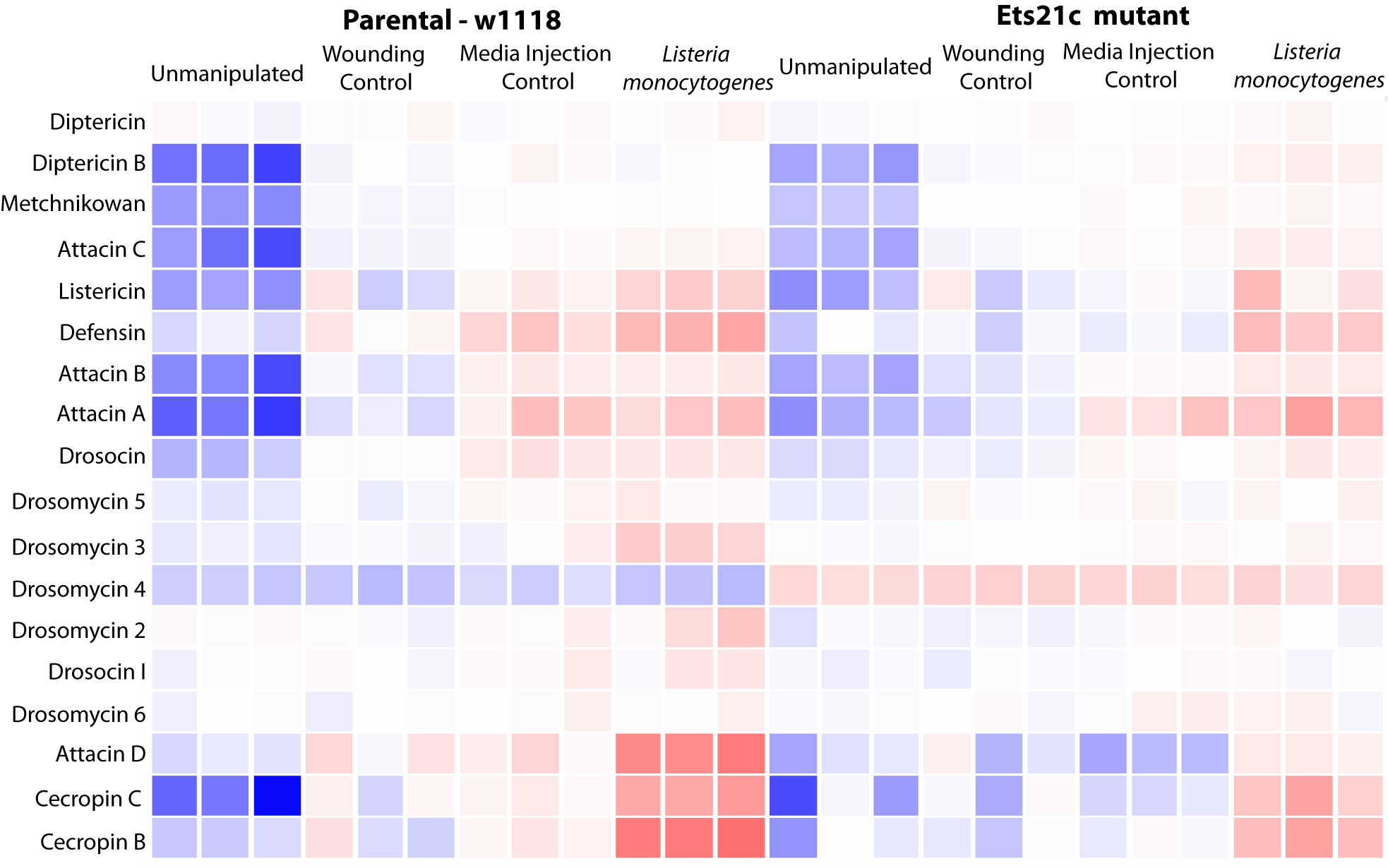

Supplement: Figure S3 — Antimicrobial peptides are induced similarly in ets21c mutants. Heatmap generated by Genespring 12.0 with a custom list of anti-microbial peptide genes. Fold changes range from −4.6 (deepest blue) to 4.6 (deepest red). (TIF) [file ppat.1002970.s004.tif]
