# Supplementary material for: How the Fly Balances Its Ability to Combat Different Pathogens
Source: PLoS Pathog. 2012 Dec 13;8(12):e1002970. doi: 10.1371/journal.ppat.1002970 (PMC3521699; doi:10.1371/journal.ppat.1002970)
Supplement: Table S1 — qRT-PCR primer sequences. Sequences of both the forward and reverse primers used for the qRT-PCR experiments. (DOCX) [file ppat.1002970.s005.docx]

Supplemental Table 1. qRT-PCR primer sequences.

| Gene | CG Number | Forward Primer | Reverse Primer |
| --- | --- | --- | --- |
| wntD | CG8458 | ATT CCC CTA GAC TCG CTG GT | CGT GTT CCT TTC CAC AAT CC |
| diptericin B | CG10794 | ACC GCA GTA CCC ACT CAA TC | CCC AAG TGC TGT CCA TAT CC |
| cecropin A1 | CG1365 | TCT TCG TTT TCG TCG CTC TC | CTT GTT GAG CGA TTC CCA GT |
| RpS15Aa | CG2033 | TGG ACC ACG AGG AGG CTA GG | GTT GGT GCA TGG TCG GTG A |
